# Efficacy and safety of direct switch to indacaterol/glycopyrronium in patients with moderate COPD: the CRYSTAL open-label randomised trial

**DOI:** 10.1186/s12931-017-0622-x

**Published:** 2017-07-18

**Authors:** Claus F. Vogelmeier, Mina Gaga, Maryam Aalamian-Mattheis, Timm Greulich, Jose M. Marin, Walter Castellani, Vincent Ninane, Stephen Lane, Xavier Nunez, Francesco Patalano, Andreas Clemens, Konstantinos Kostikas, on behalf of the CRYSTAL study investigators

**Affiliations:** 1 Department of Medicine, Pulmonary and Critical Care Medicine, University Medical Centre Giessen and Marburg, Philipps-Universität Marburg, Member of the German Centre for Lung Research (DZL), Marburg, Germany; 2 7th Respiratory Medicine Department, Athens Chest Hospital Sotiria, Athens, Greece; 3 Novartis Pharma AG, Basel, Switzerland; 4 Respiratory Medicine, Hospital Universitario Miguel Servet, Zaragoza, Spain; 5 Centro de Investigación Biomédica en Red de Enfermedades Respiratorias (CIBERES), Madrid, Spain; 6 Department of Respiratory Physiopathology, Palagi Hospital, Florence, Italy; 7 CHU Saint-Pierre - Service de Pneumologie, Brussells, Belgium; 8 Adelaide & Meath Hospital, Dublin, Ireland; 9 TFS Develop, Barcelona, Spain

**Keywords:** Chronic obstructive pulmonary disease, Dual bronchodilation, Indacaterol/glycopyrronium, Direct switch, Open-label

## Abstract

**Background:**

Dual bronchodilation combining a long-acting β_2_-agonist (LABA) and a long-acting muscarinic antagonist (LAMA) is the preferred choice of treatment recommended by the Global Initiative for Chronic Obstructive Lung Disease (GOLD) 2017 guidelines for the management of patients with moderate-to-severe chronic obstructive pulmonary disease (COPD). The once-daily (q.d.) fixed-dose combination (FDC) of LABA, indacaterol 110 μg and LAMA, glycopyrronium 50 μg (IND/GLY 110/50 μg q.d.) demonstrated superior improvements in lung function, dyspnoea and overall health status and better tolerability against LABA or LAMA monotherapies and combination of LABA and inhaled corticosteroid (ICS) in more than 11,000 patients with moderate-to-severe COPD in several randomised controlled clinical trials.

**Methods:**

The CRYSTAL study was the first, 12-week, randomised, open-label trial that evaluated the efficacy and safety of a direct switch from previous treatments to IND/GLY 110/50 μg q.d. on lung function and dyspnoea in patients with moderate COPD and a history of up to one exacerbation in the previous year. Patients were divided into 2 groups according to their background therapy and symptom scores and were randomised (3:1) to IND/GLY or to continue with their previous treatments.

**Results:**

The study included 4389 randomised patients, of whom 2160 were in groups switched to IND/GLY (intention-to-treat population). The effect of IND/GLY was superior to LABA + ICS on trough forced expiratory volume in 1 s (FEV_1_; treatment difference, Δ = +71 mL) and transition dyspnoea index (TDI; [Δ = 1.09 units]), and to LABA or LAMA on trough FEV_1_ (Δ = +101 mL) and a TDI (Δ = 1.26 units). Improvements in health status and lower rescue medication use were also observed with IND/GLY. The safety profile of the study medication was similar to that observed in previous studies.

**Conclusions:**

IND/GLY demonstrated superior improvements in lung function and dyspnoea after direct switch from previous treatments.

**Trial registration:**

ClinicalTrials.gov number: NCT01985334.

**Electronic supplementary material:**

The online version of this article (doi:10.1186/s12931-017-0622-x) contains supplementary material, which is available to authorized users.

## Background

Maintenance inhaled treatment of chronic obstructive pulmonary disease (COPD) is based on long-acting β_2_-agonist (LABA) and/or long-acting muscarinic antagonists (LAMA) and LABA/inhaled corticosteroid (ICS) [1]. Despite treatment, most of the patients remain symptomatic, requiring a change in therapy [2].

A once-daily (q.d.) combination of indacaterol and glycopyrronium (IND/GLY) was effective and well tolerated in patients with moderate-to-severe COPD in several phase III trials [3, 4]. Such pivotal trials are usually performed in selected patients and hospital settings [5]. Patients with COPD treated in primary care settings often differ from those included in large randomised controlled trials (RCTs) [6]. Moreover, in RCTs, prior to randomisation, patients typically go through a washout period, in contrast to clinical practice where treatment changes occur without any washout period [5]. The effectiveness of a treatment in clinical practice can be evaluated more appropriately in a direct switch study in primary care settings, engaging a relevant patient pool with less stringent inclusion criteria [6].

CRYSTAL (effect of glyCopyrronium or indacateRol maleate and glYcopyrronium bromide fixed-dose combination [FDC] on SympToms and heALth status in patients with moderate COPD) was a 12-week, prospective, multi-centre, open-label study carried out in clinical practice settings to evaluate the impact of a direct switch to GLY or IND/GLY from previous standard-of-care treatments on lung function and dyspnoea in symptomatic patients with moderate COPD.

In this manuscript, we focus on the fully recruited and adequately powered groups directly switched to IND/GLY; the results of the GLY groups are described in the Additional file 1: Section S6, Tables S1, S2, S3 and S4 and Figures S5 and S6.

## Methods

### Study design and patients

**Fig. 1:**
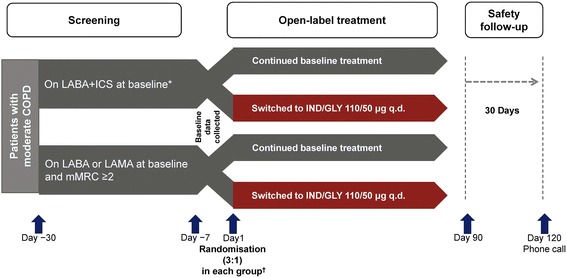
CRYSTAL study design (groups switched to IND/GLY). *Free or fixed-dose combination. ^†^Randomisation ratio (switched: baseline treatments) = 3:1 by stratifying background medications. All comparisons were for superiority of the switched treatment. COPD, chronic obstructive pulmonary disease; ICS, inhaled corticosteroids; IND/GLY, indacaterol/glycopyrronium; LABA, long-acting β_2_-agonist; LAMA, long-acting muscarinic antagonist; mMRC, modified Medical Research Council; q.d., once daily; SABA, short-acting β_2_-agonist; SAMA, short-acting muscarinic antagonist

CRYSTAL was a prospective, multi-centre, 12-week, randomised, open-label, pragmatic study, designed to evaluate the effectiveness and safety of GLY or IND/GLY after a direct switch from previous treatments in clinical settings in patients with moderate COPD. The study comprised a 30-day safety follow-up period, conducted from June 2014 to April 2016 at 560 sites across 23 countries in Europe, in both hospital and primary care settings (Fig. 1 and Additional file 1: Figure S1).

CRYSTAL included patients aged ≥40 years with a clinical diagnosis of COPD [7], current or ex-smokers with a smoking history of ≥10 pack-years, a modified Medical Research Council (mMRC) score ≥1, a moderate post-bronchodilator airflow limitation (forced expiratory volume in 1 s [FEV_1_] ≥50% and <80% predicted and FEV_1_/forced vital capacity (FVC) ratio <0.7) and patients who have been on a stable dose of baseline treatment with any short-acting β_2_-agonists (SABA) and/or short-acting muscarinic antagonists (SAMA) or LABA or LAMA or LABA + ICS in free or FDCs for at least 3 months before screening. Patients who had a body mass index (BMI) >40 kg/m^2^, active/clinical history of asthma, narrow-angle glaucoma, urinary retention or severe renal impairment, malignancy of any organ system in the past 5 years, any cardiac disorders (myocardial infarction, arrhythmia, etc.), treatment with non-selective β-blockers, hypersensitivity to any of the investigational drugs or their constituents used in this study, and ≥2 COPD exacerbations requiring systemic corticosteroids and/or antibiotics or with ≥1 hospitalisation in the previous 12 months were excluded from the study. The detailed inclusion and exclusion criteria can be found in the Additional file 1: Sections S1.1 and S1.2.

Patients were randomised (3:1) based on previous treatments and mMRC scores to either a direct switch to GLY 50 μg or IND/GLY 110/50 μg q.d. or to remain on their baseline therapy as active control (Fig. 1 and Additional file 1: Figure S1). The study was sponsored by Novartis and was performed according to the Declaration of Helsinki. All patients provided written informed consent (ClinicalTrials.gov number: NCT01985334).

During the course of the study, recruitment of patients switched to GLY was significantly lower than expected. Therefore, recruitment in these groups was stopped at the time of completion of randomisation in the groups of patients switched to IND/GLY. Analysis was performed in all study groups, but GLY groups were underpowered. In this manuscript, we focus on the fully recruited and adequately powered groups directly switched to IND/GLY; the results of GLY groups are described in the Additional file 1: Section S6, Tables S1, S2, S3 and S4 and Figures S5 and S6.

### Study objectives

The co-primary endpoints of the study were (i) superiority of IND/GLY against LABA or LAMA and LABA + ICS; (ii) superiority of GLY versus SABA and/or SAMA and (iii) non-inferiority of GLY versus LABA or LAMA, in terms of improvements in trough FEV_1_ (average of measurements at 45 and 15 min before dosing) and transition dyspnoea index (TDI) total score at Week 12. Secondary objectives included change from baseline in the total score of COPD assessment test (CAT), clinical COPD questionnaire (CCQ), use of rescue medications (number of puffs/day and days without rescue medication [salbutamol] use) and safety and tolerability of study treatments. TDI, CAT and CCQ assessments were performed in accordance with standard protocols [8–10]. All patients recruited in the CRYSTAL study received an electronic diary (e-diary) for capturing their daily symptoms and frequency of rescue medication use. Additional details on the assessment can be found in the Additional file 1 : Section S1.4.

### Statistical analysis

The intention-to-treat (ITT) population included all randomised patients who received at least one dose of the study medication, whereas, the per-protocol (PP) population comprised all patients in the ITT population with valid measurements of the primary endpoints without major protocol deviations (categories of reportable protocol deviations are listed in the Additional file 1). Efficacy analyses were performed on both ITT and PP populations (primary endpoint only for the latter). For the two co-primary endpoints (trough FEV_1_ and TDI after 12 weeks of treatment), a mixed model with treatment as a fixed effect and baseline value as a covariate was carried out. The primary efficacy endpoints were analysed for each group separately, based on the number of patients recruited in each treatment group and their respective baseline values, without accounting for an overall effect in all groups. Hence, evaluation of co-primary endpoints was done separately for GLY and IND/GLY groups. In addition, the proportion of responders, i.e. patients reaching a minimal clinically significant change from baseline (100 mL in trough FEV_1_; 1 unit in TDI) at 12 weeks, was presented and analysed using the observed odds ratio (OR) for responders versus non-responders. Secondary efficacy endpoints (CAT total score, CCQ total score, number of puffs of rescue medication and patient-reported symptoms of COPD) were analysed at Week 12 using the Mann–Whitney–Wilcoxon test. The proportion of CAT and CCQ responders (defined as a significant change from baseline of −2 and −0.4 units, respectively) were also presented. All safety analyses were performed on the safety population, consisting of all patients who received at least one dose of the study treatment.

## Results

### Demographics and baseline characteristics

Of the 4389 patients randomised in the study, 2160 patients were included in groups that switched to IND/GLY (baseline treatment with LABA + ICS: *n* = 1083 [ITT population] and *n* = 791 [PP population] and baseline treatment with LABA or LAMA: *n* = 1077 [ITT population] and *n* = 873 [PP population]). Patient disposition in all groups is presented in the Additional file 1: Figure S2. Demographic and baseline characteristics of patients randomised to IND/GLY and comparators are summarised in Table 1. Differences in mMRC scores and baseline medication between groups switched to IND/GLY from LABA or LAMA compared with the patients switched from LABA + ICS are explained by protocol-defined inclusion criteria, with most patients who switched to IND/GLY from baseline LABA or LAMA having an mMRC score ≥ 2. Demographic information and baseline characteristics of groups switched to GLY are presented in the Additional file 1: Table S1.

**Table 1:** Demographics and baseline characteristics of patients who switched to IND/GLY (ITT population)

Characteristics	LABA + ICS *n* = 269	IND/GLY *n* = 811	LABA or LAMA^b^ *n* = 268	IND/GLY^b^ *n* = 811
Age, years	64.4 (8.9)	64.6 (8.7)	65.2 (7.6)	65.4 (8.3)
Gender – male, *n* (%)	164 (61.0%)	528 (65.1%)	176 (65.7%)	537 (66.2%)
Current smoker, *n* (%)	138 (51.3%)	392 (48.3%)	135 (50.4%)	435 (53.6%)
Post-bronchodilator FEV_1_, L	1.76 (0.5)	1.80 (0.5)	1.76 (0.4)	1.79 (0.5)
Post-bronchodilator FEV_1_, % predicted of normal value	63.3 (8.3)	63.7 (8.7)	63.5 (8.2)	63.8 (8.8)
Dyspnoea – mMRC grade, *n* (%)				
0	1 (0.4%)	11 (1.4%)	2 (0.8%)	2 (0.3%)
1	138 (51.3%)	435 (53.6%)	28 (10.4%)	63 (7.8%)
≥ 2	129 (48.0%)	365 (45.0%)	238 (88.8%)	745 (91.9%)
Number of exacerbation in the previous 12 months, *n* (%)				
0	193 (71.8%)	587 (72.4%)	212 (79.1%)	671 (82.7%)
1	72 (26.8%)	220 (27.1%)	56 (20.9%)	136 (16.8%)
≥ 2	4 (1.5%)	4 (0.5%)	0 (0.0%)	4 (0.5%)
Baseline treatments, *n* (%)^a^				
LAMA (only monotherapy)	-	2 (0.2%)	138 (51.3%)	445 (55.1%)
LABA (only monotherapy)	-	3 (0.4%)	125 (46.5%)	341 (42.2%)
LABA + ICS (free or fixed-dose combination)	266 (98.9%)	787 (96.7%)	-	2 (0.3%)
Others^c^	12 (4.4%)	39 (4.8%)	12 (4.5%)	44 (5.5%)

^a^At baseline, some of the patients were receiving more than one type of COPD medication

^b^Patients had an mMRC score ≥ 2

^c^ICS, systemic corticosteroids, methylxanthines, roflumilast etc

Data are presented as mean (standard deviation), unless otherwise stated

COPD, chronic obstructive pulmonary disease; FEV_1_, forced expiratory volume in 1 s; IND/GLY, indacaterol/glycopyrronium; ITT, intention-to-treat; LABA + ICS, long-acting β_2_-agonist + inhaled corticosteroid; LAMA, long-acting muscarinic antagonist; mMRC, modified Medical Research Council

### Impact of a direct switch to IND/GLY on lung function and dyspnoea (co-primary endpoints)

**Fig. 2:**
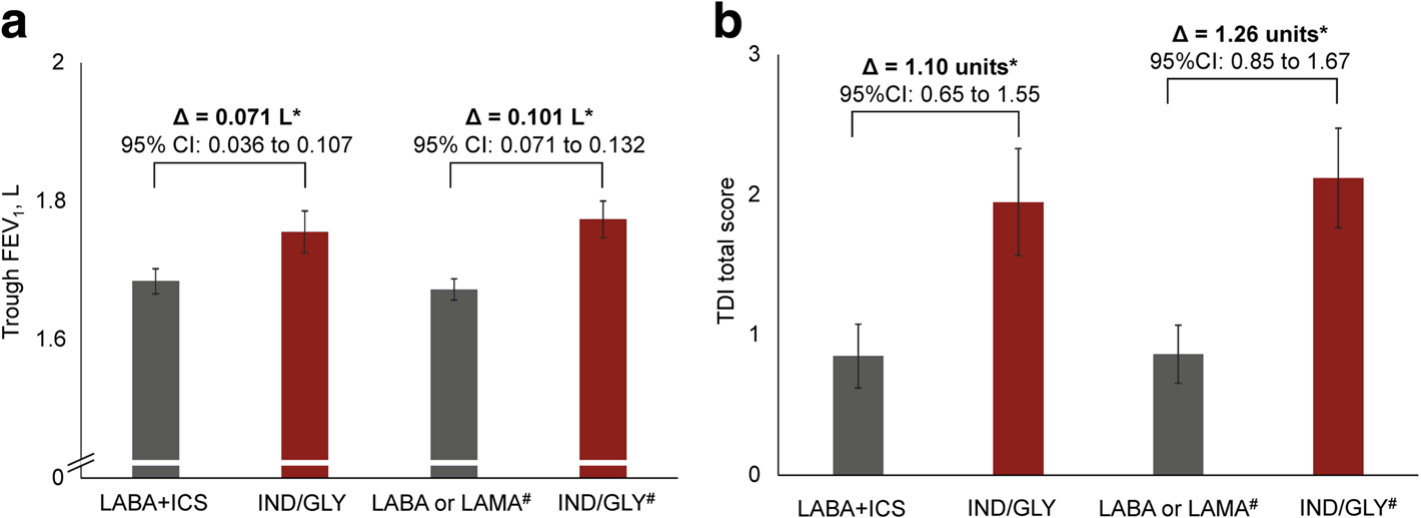
Change from baseline in (**a**) trough FEV_1_ and (**b**) TDI total scores with IND/GLY versus comparators at Week 12 (ITT population). *P < 0.0001. ^#^Patients had an mMRC score ≥ 2. Data are least squares means (95% CI). ∆, treatment difference; CI, confidence interval; FEV_1_, forced expiratory volume in 1 s; IND/GLY, indacaterol/glycopyrronium; ITT, intention-to-treat; LABA + ICS, long-acting β_2_-agonist + inhaled corticosteroid; LAMA, long-acting muscarinic antagonist; mMRC, modified Medical Research Council; TDI, transition dyspnoea index

In the ITT population, IND/GLY provided superior improvement in trough FEV_1_ at Week 12 versus LABA + ICS (treatment difference [Δ] = +71 mL, *P* < 0.0001) and versus LABA or LAMA monotherapies (Δ = +101 mL, *P* < 0.0001; Fig. 2a). Similar improvements were observed in the PP population (versus LABA + ICS: Δ = +85 mL, *P* < 0.0001; and versus LABA or LAMA: Δ = +105 mL, *P* < 0.0001).

IND/GLY also provided superior improvement in TDI total score at Week 12 versus LABA + ICS (Δ = 1.10 units, *P* < 0.0001) and versus LABA or LAMA monotherapies (Δ = 1.26 units, *P* < 0.001; Figure 2B) in the ITT population. Similar improvements were observed in the PP population (versus LABA + ICS: Δ = 1.33 units, *P* < 0.0001; and versus LABA or LAMA monotherapies: Δ = 1.26 units, *P* < 0.0001).

A significantly higher proportion of patients reached the minimal clinically important difference (MCID) of 100 mL for trough FEV_1_ in the IND/GLY groups compared with LABA + ICS groups (OR: 1.90; 95% confidence interval [CI]: 1.42 to 2.55) and compared with LABA or LAMA (OR: 2.53; 95% CI: 1.86 to 3.42; Table 2). A significantly higher proportion of patients in the IND/GLY groups had reached the MCID for TDI total score of ≥1 unit compared with LABA + ICS (OR: 2.61; 95% CI: 1.94 to 3.50) and LABA or LAMA (OR: 2.85; 95% CI: 2.13 to 3.82; Table 2). IND/GLY demonstrated consistent improvement in lung function and dyspnoea compared with LABA or LAMA and LABA + ICS in both ITT and PP populations during the 12 weeks.

**Table 2:** Effects of direct switch to IND/GLY from baseline treatments on trough FEV_1_ and TDI total scores: proportion of patients achieving MCID (ITT population)

	LABA + ICS *n* = 269	IND/GLY *n* = 811	LABA OR LAMA^a^ *n* = 268	IND/GLY^a^ *n* = 811
Patients achieved MCID in trough FEV_1_ (≥100 mL difference from baseline)	90 (33.4%)	379 (46.7%)	74 (27.6%)	388 (47.8%)
Trough FEV_1_ responders (improvement ≥100 mL; OR [95% CI])	1.90 (1.42 to 2.55)		2.53 (1.86 to 3.42)	
Patients achieved MCID in TDI total scores (≥1-unit difference from baseline)	91 (33.8%)	427 (52.7%)	95 (35.5%)	466 (57.5%)
TDI responders (improvement ≥1 unit; OR [95% CI])	2.61 (1.94 to 3.50)		2.85 (2.13 to 3.82)	

^a^Patients had an mMRC score ≥ 2

Data are presented as n (%), unless otherwise stated.CI, confidence interval; FEV_1_, forced expiratory volume in 1 s; IND/GLY, indacaterol/glycopyrronium; ITT, intention-to-treat; LABA + ICS, long-acting β_2_-agonist + inhaled corticosteroid; LAMA, long-acting muscarinic antagonist; MCID, minimal clinically important difference; OR, odds ratio; TDI, transition dyspnoea index

In post-hoc subgroup analyses, IND/GLY provided consistent improvements in trough FEV_1_ versus LABA + ICS or LABA or LAMA in all subgroups of patients categorised according to age, sex, smoking status, history of exacerbations in the previous year, baseline trough FEV_1_, bronchodilator reversibility, mMRC score or baseline treatments (Additional file 1: Figure S3A and B). Moreover, IND/GLY provided consistent improvements in TDI versus LABA + ICS or LABA or LAMA in all subgroups of patients categorised according to the same baseline characteristics (Additional file 1: Figure S4A and B).

### Impact of direct switch to IND/GLY on health status and rescue medication use

Numerical improvements from baseline in the total CAT score were observed at Week 12 with IND/GLY versus the comparator treatments; however, these differences did not reach statistical significance (Table 3). Statistically significant improvements in the CCQ total scores from baseline were observed at Week 12 with IND/GLY versus the comparator treatments. More patients on IND/GLY reached the MCID of 2-unit improvement in CAT with IND/GLY versus LABA + ICS (OR: 1.44; 95% CI: 1.06 to 1.95) and LABA or LAMA monotherapies (OR: 1.12; 95% CI: 0.83 to 1.50; Table 3).

**Table 3:** Effects of a direct switch to IND/GLY from baseline treatments on CAT, CCQ and rescue medication use (ITT population)

	LABA + ICS *n* = 269	IND/GLY *n* = 811	LABA or LAMA^a^ *n* = 268	IND/GLY^a^ *n* = 811
Total CAT score, change from baseline at Week 12	−0.4 (4.8)	−1.4 (5.4)	−0.9 (5.0)	−1.9 (5.3)
Patients who achieved MCID in total CAT score (≥2 units difference from baseline), *n* (%)	89 (33.1%)	311 (38.4%)	112 (41.8%)	351 (43.3%)
CAT responders (decrease ≥2 units; OR [95% CI])	1.44 (1.06 to 1.95)		1.12 (0.83 to 1.50)	
Total CCQ score, change from baseline at Week 12	−0.1 (0.7)	−0.2 (0.8)*	−0.1 (0.8)	−0.3 (0.8)***
Patients who achieved MCID in the total CCQ score (≥0.4 units difference from baseline), *n* (%)	64 (23.8%)	243 (30.0%)	74 (27.6%)	293 (36.1%)
CCQ responders (decrease ≥0.4 units; OR [95% CI])	1.53 (1.10 to 2.12)		1.58 (1.16 to 2.17)	
Number of puffs of rescue medication over 12 weeks	1.6 (1.7)	1.1 (1.4)****	1.4 (1.4)	1.1 (1.3)***
Percentage of days without rescue medication use over 12 weeks	41.7 (42.9)	49.9 (43.4)**	38.8 (42.6)	46.7 (42.6)**

*P < 0.05; **P < 0.01; ***P < 0.001; ****P < 0.0001

^a^Patients had an mMRC score ≥ 2

Data are presented as mean (stadard deviation), unless otherwise stated

CAT, COPD assessment test; CCQ, clinical COPD questionnaire; CI, confidence interval; COPD, chronic obstructive pulmonary disease; IND/GLY, indacaterol/glycopyrronium; ITT, intention-to-treat; LABA + ICS, long-acting β_2_-agonist + inhaled corticosteroid; LAMA, long-acting muscarinic antagonist; MCID, minimal clinically important difference; mMRC, modified Medical Research Council; OR, odds ratio

Patients switched to IND/GLY presented lower rescue medication use (puffs/day) and more days without rescue medication use compared with patients who continued on LABA + ICS or LABA or LAMA (Table 3).

### Safety

The safety profile of GLY and IND/GLY was comparable to that of comparators (Table 4 and Additional file 1: Table S5). There were numerical differences between IND/GLY and the continued baseline treatments. However, there were no differences in severe or fatal adverse events between the various treatment groups, and no new safety signals related to any treatment were identified in this study. Four deaths, two in each group, those switched to IND/GLY from LABA or LAMA and those who remained on LABA or LAMA, were reported during the study, and none were considered to be treatment related.

**Table 4:** Treatment-emergent adverse events and serious adverse events during the study period (safety set)

	LABA + ICS *n* = 269	IND/GLY *n* = 816	LABA or LAMA^a^ *n* = 269	IND/GLY^a^ *n* = 814
Any adverse event	56 (20.8%)	235 (28.8%)	58 (21.6%)	221 (27.2%)
Any serious adverse event	6 (2.2%)	22 (2.7%)	10 (3.7%)	34 (4.2%)
Any suspected drug-related adverse event	2 (0.7%)	52 (6.4%)	2 (0.7%)	34 (4.2%)
Any suspected drug-related serious adverse event	1 (0.4%)	1 (0.1%)	0 (0.0%)	0 (0.0%)
Any adverse event leading to treatment withdrawal	2 (0.7%)	22 (2.7%)	3 (1.1%)	26 (3.2%)
Any adverse event with a fatal outcome (death)	0 (0.0%)	0 (0.0%)	2 (0.7%)	2 (0.3%)

^a^Patients had an mMRC score ≥ 2

Data are presented as number of incidences (%)

IND/GLY, indacaterol/glycopyrronium; ITT, intention-to-treat; LABA + ICS, long-acting β_2_-agonist + inhaled corticosteroid; LAMA, long-acting muscarinic antagonist; mMRC, modified Medical Research Council

## Discussion

In the randomised, open-label CRYSTAL study in patients with moderate COPD, a direct switch to IND/GLY resulted in significant improvements in lung function and dyspnoea compared with continuation of LABA or LAMA monotherapies and LABA + ICS combinations. Patients on IND/GLY also achieved improvements in health status and presented reduced rescue medication use, with a safety profile similar to that reported in RCTs.

This is, to the best of our knowledge, the first study to evaluate the effectiveness of a step-up strategy from single long-acting bronchodilators to a dual bronchodilator. This is in accordance with the recent Global Initiative for Chronic Obstructive Lung Disease (GOLD) 2017 strategy document that suggests escalation to dual bronchodilation in patients who have high level of symptoms and low risk of developing exacerbations (GOLD group B) and remain symptomatic on a LABA or LAMA [1]. Existing evidence supports the de-escalation of appropriate stable COPD patients from triple therapy (LABA + LAMA + ICS) to dual bronchodilation [11, 12], but data supporting treatment escalation are lacking. The CRYSTAL study supports the GOLD step-up recommendation from a single bronchodilator regimen to IND/GLY in symptomatic patients [1].

Although the efficacy and safety of IND/GLY has been demonstrated in well-designed explanatory trials, real-world evidence is currently missing. In pragmatic trials conducted in clinical practice settings, outcome measures are typically clinically-relevant assessments that may support clinicians to decide between a new intervention and previously available treatments [5]. The use of less stringent inclusion and exclusion criteria and direct switching between treatments without a washout period mimics the way treatment changes occur in clinical practice [5]. The CRYSTAL study implemented a direct switch from various available treatments in symptomatic patients with moderate COPD, without a washout period. In the CRYSTAL study, patients were switched from a particular treatment to IND/GLY and patients continuing baseline therapy (LABA + ICS or LABA or LAMA) served as the control group. The study was conducted in 23 European countries and included approximately 50% non–hospital-based investigators (office-based pulmonologists and general practitioners), thus representing a wide range of clinical practice settings.

The efficacy and safety results of IND/GLY in this study were consistent with previous explanatory trials of the IGNITE programme, consisting of 11 trials conducted in >11,000 patients with moderate-to-very severe COPD [3, 4, 13]. In the 26-week ILLUMINATE study, IND/GLY demonstrated superior improvements in the trough FEV_1_ of 103 mL [3], whereas in the 1-year FLAME study, the Δ was 62 mL compared with LABA/ICS [4]. Similarly, in the 26-week SHINE study, IND/GLY showed greater improvements in lung function in trough FEV_1_, with a Δ versus its monocomponents IND and GLY of 70 mL and 90 mL, respectively and 80 mL versus the open-label tiotropium [14]. IND/GLY also demonstrated a significant improvement in TDI versus salmeterol-fluticasone in the ILLUMINATE study (Δ = 0.76 units) and versus LABA or LAMA (e.g. with a Δ of 0.51 units versus open-label tiotropium in the SHINE study [3] and 0.49 units versus blinded tiotropium in the 6-week BLAZE study [15]). Studies of other available LABA/LAMA FDCs (e.g. aclidinium/formoterol and umeclidinium/vilanterol) have reported significant improvements in dyspnoea in comparison with baseline or placebo, but in some of these studies, non-significant differences were also observed when compared with active comparators such as LAMA or LABA/ICS [16–19].

In the CRYSTAL study, IND/GLY showed greater improvements in TDI total score versus the comparators than those observed in previous RCTs. The open-label comparison of a new and effective drug versus previous treatments may have resulted in a more pronounced effect on TDI total score. The inclusion of objective endpoints has been strongly recommended in open-label trials as they are less prone to bias [20], and therefore FEV_1_ was a pre-specified co-primary endpoint in this study. The improvements in dyspnoea (TDI) and lung function (trough FEV_1_) in CRYSTAL were concurrent and the magnitude of the effect on lung function was similar to that shown in RCTs. Based on the above facts, we believe that the greater improvement in TDI total score, although higher than that observed in RCTs, is not a chance finding.

A higher numerical reduction in CAT total score with IND/GLY versus both LABA + ICS and LABA or LAMA monotherapy was observed that did not reach statistical significance. In contrast, there was a statistically significant improvement in the CCQ total score versus both comparators. A potential explanation for this discrepancy is that different scores have different sensitivities in the response to treatment. Moreover, CAT is a health status tool that was developed to help patients and their clinicians assess and quantify the symptoms and impacts of COPD [21] and has been shown so far to be responsive to changes in health status following exacerbations and to pulmonary rehabilitation [22]. Further studies are needed to evaluate the responsiveness of different health status tools to pharmacotherapy options.

The overall incidence of adverse events was similar in the investigational and comparator treatment groups, with a slightly higher percentage in IND/GLY groups. This difference may be attributed to a potential reporting bias of adverse events against the newly initiated treatment (IND/GLY) compared with previous treatments that have already been well tolerated by the patients. In open-label trials, there is a risk of increased adverse event reporting, both from patients who may research the ‘new’ drug and may be influenced in their reporting behaviour, as well as from the investigators who may be more susceptible to report adverse events related to the new drug [23]. Importantly, the overall incidence of severe and fatal adverse events was similar in the IND/GLY and comparator treatments.

Improvements in lung function, symptoms, quality of life and exercise tolerance represent major needs for patients with COPD. Although we did not evaluate the exercise capacity of patients in the CRYSTAL study, the improvement in breathlessness and health status may resonate in improvement of exercise tolerance in certain patients [24]. Long-acting bronchodilators also act as lung deflators, which may lead to the improvement in static and dynamic hyperinflation [25]. IND/GLY has demonstrated significant improvements in inspiratory capacity, dyspnoea, exercise capacity and other patient-reported outcomes in RCTs [3, 4, 24, 26, 27], and the consistent results of the CRYSTAL study support that these beneficial effects may be experienced also in routine clinical practice.

The CRYSTAL study was appropriately designed to complement previous RCTs by providing information on a direct switch to IND/GLY under routine clinical practice conditions, but, as other open-label studies, may have certain limitations. Firstly, some of the non-frequently exacerbating patients with moderate COPD recruited in the CRYSTAL study were appropriately treated with a single long-acting bronchodilator, whereas most of the patients on LABA + ICS were inappropriately treated according to the GOLD recommendations [28], as it happens often in clinical practice. Importantly, the CRYSTAL study results show that even in these mildly symptomatic patients, the switch from a mono-bronchodilator regimen (with or without ICS) to a dual bronchodilator (IND/GLY) significantly improved their lung function, breathlessness and overall quality of life. Secondly, the duration of the study was only 12 weeks, and this short time frame does not allow for a clear understanding of the long-term effectiveness of IND/GLY and its potential effects on exacerbations and long-term safety. Nevertheless, the consistency of the CRYSTAL study results with those of previous RCTs indicates that the effectiveness of IND/GLY in clinical settings is likely to be similar to the efficacy observed in RCTs. Moreover, the direct switch from a LABA + ICS regimen may raise a question on carry-over effect of pre-treatments; however, given the short half-life of ICS, a period of 12 weeks is long enough to make any carry-over effects of ICS treatment unlikely. The third limitation is the fact that it was an open-label study with a potential reporting bias regarding patient-reported outcomes, e.g. measures of dyspnoea and health-related quality of life. However, in order to avoid a selection bias in this open-label study setting, patients were randomised at baseline. Finally, the short study duration does not allow for appropriate evaluation of exacerbation prevention in this setting. The results of the CRYSTAL study provide reassurance on the efficacy and safety of dual bronchodilation compared with mono-bronchodilators or LABA + ICS combinations.

## Conclusion

In the CRYSTAL study, a direct switch to IND/GLY demonstrated significant improvements in lung function and dyspnoea after 12 weeks of treatment in symptomatic patients with moderate COPD compared to the continuation of previous treatment with LABA or LAMA or LABA + ICS.

## Additional file

Additional file 1: Supplementary file for the CRYSTAL study. (DOCX 984 kb)

## Abbreviations

BMI: body mass index
CAT: COPD assessment test
CCQ: clinical COPD questionnaire
CI: confidence interval
COPD: chronic obstructive pulmonary disease
e-diary: electronic diary
FDC: fixed-dose combination
FEV_1_: forced expiratory volume in 1 s
FVC: forced vital capacity
GLY: glycopyrronium
GOLD: Global Initiative for Chronic Obstructive Lung Disease
ICS: inhaled corticosteroid
IEC: Independent Ethics Committee
IND/GLY: indacaterol/glycopyrronium
IRB: Institutional Review Board
ITT: intention to treat
LABA: long-acting β_2_-agonist
LAMA: long-acting muscarinic antagonist
MCID: minimal clinically important difference
mMRC: modified Medical Research Council
OR: odds ratio
PP: per protocol
q.d.: once daily
RCT: randomised controlled trial
SABA: short-acting β_2_-agonist
SAMA: short-acting muscarinic antagonist
TDI: transition dyspnoea index
Δ: treatment difference

## References

[1] Global Strategy for the Diagnosis, Management and Prevention of COPD, Global Initiative for Chronic Obstructive Lung Disease (GOLD) 2017. Available from www.goldcopd.org. Accessed on 4 May 2017

[2] Dransfield MT, Bailey W, Crater G, Emmett A, O'Dell DM, Yawn B (2011). Disease severity and symptoms among patients receiving monotherapy for COPD. Prim Care Respir J.

[3] Vogelmeier CF, Bateman ED, Pallante J, Alagappan VK, D'Andrea P, Chen H, Banerji D (2013). Efficacy and safety of once-daily QVA149 compared with twice-daily salmeterol-fluticasone in patients with chronic obstructive pulmonary disease (ILLUMINATE): a randomised, double-blind, parallel group study. Lancet Respir Med.

[4] Wedzicha JA, Banerji D, Chapman KR, Vestbo J, Roche N, Ayers RT, Thach C, Fogel R, Patalano F, Vogelmeier CF, FLAME Investigators (2016). Indacaterol-Glycopyrronium versus Salmeterol-Fluticasone for COPD. N Engl J Med.

[5] Ford I, Norrie J (2016). Pragmatic trials. N Engl J Med.

[6] Kruis AL, Stallberg B, Jones RC, Tsiligianni IG, Lisspers K, van der Molen T, Kocks JW, Chavannes NH (2014). Primary care COPD patients compared with large pharmaceutically-sponsored COPD studies: an UNLOCK validation study. PLoS One.

[7] Global Strategy for the Diagnosis, Management and Prevention of COPD, Global Initiative for Chronic Obstructive Lung Disease (GOLD) 2013. Available from www.goldcopd.org.

[8] Gupta N, Pinto LM, Morogan A, Bourbeau J (2014). The COPD assessment test: a systematic review. Eur Respir J.

[9] Kon SS, Dilaver D, Mittal M, Nolan CM, Clark AL, Canavan JL, Jones SE, Polkey MI, Man WD (2014). The clinical COPD questionnaire: response to pulmonary rehabilitation and minimal clinically important difference. Thorax.

[10] Mahler DA, Waterman LA, Ward J, McCusker C, ZuWallack R, Baird JC (2007). Validity and responsiveness of the self-administered computerized versions of the baseline and transition dyspnea indexes. Chest.

[11] Magnussen H, Disse B, Rodriguez-Roisin R, Kirsten A, Watz H, Tetzlaff K, Towse L, Finnigan H, Dahl R, Decramer M (2014). Withdrawal of inhaled glucocorticoids and exacerbations of COPD. N Engl J Med.

[12] Watz H, Tetzlaff K, Wouters EF, Kirsten A, Magnussen H, Rodriguez-Roisin R, Vogelmeier C, Fabbri LM, Chanez P, Dahl R (2016). Blood eosinophil count and exacerbations in severe chronic obstructive pulmonary disease after withdrawal of inhaled corticosteroids: a post-hoc analysis of the WISDOM trial. Lancet Respir Med.

[13] Horita N, Kaneko T (2015). Role of combined indacaterol and glycopyrronium bromide (QVA149) for the treatment of COPD in Japan. Int J Chron Obstruct Pulmon Dis.

[14] Bateman ED, Ferguson GT, Barnes N, Gallagher N, Green Y, Henley M, Banerji D (2013). Dual bronchodilation with QVA149 versus single bronchodilator therapy: the SHINE study. Eur Respir J.

[15] Mahler DA, Decramer M, D’Urzo A, D'Urzo A, Worth H, White T, Alagappan VK, Chen H, Gallagher N, Kulich K, Banerji D (2014). Dual bronchodilation with QVA149 reduces patient-reported dyspnoea in COPD: the BLAZE study. Eur Respir J.

[16] Decramer M, Anzueto A, Kerwin E, Kaelin T, Richard N, Crater G, Tabberer M, Harris S, Church A (2014). Efficacy and safety of umeclidinium plus vilanterol versus tiotropium, vilanterol, or umeclidinium monotherapies over 24 weeks in patients with chronic obstructive pulmonary disease: results from two multicentre, blinded, randomised controlled trials. Lancet Respir Med.

[17] Donohue JF, Niewoehner D, Brooks J, O'Dell D, Church A (2014). Safety and tolerability of once-daily umeclidinium/vilanterol 125/25 mcg and umeclidinium 125 mcg in patients with chronic obstructive pulmonary disease: results from a 52-week, randomized, double-blind, placebo-controlled study. Respir Res.

[18] Singh D, Jones PW, Bateman ED, Korn S, Serra C, Molins E, Caracta C, Gil EG, Leselbaum A (2014). Efficacy and safety of aclidinium bromide/formoterol fumarate fixed-dose combinations compared with individual components and placebo in patients with COPD (ACLIFORM-COPD): a multicentre, randomised study. BMC Pulm Med.

[19] Vogelmeier C, Paggiaro PL, Dorca J, Sliwinski P, Mallet M, Kirsten AM, Beier J, Seoane B, Segarra RM, Leselbaum A (2016). Efficacy and safety of aclidinium/formoterol versus salmeterol/fluticasone: a phase 3 COPD study. Eur Respir J 2016.

[20] Beeh KM, Beier J, Donohue JF (2012). Clinical trial design in chronic obstructive pulmonary disease: current perspectives and considerations with regard to blinding of tiotropium. Respir Res.

[21] Jones PW, Brusselle G, Dal Negro RW, Ferrer M, Kardos P, Levy ML, Perez T, Soler Cataluna JJ, van der Molen T, Adamek L, Banik N (2011). Properties of the COPD assessment test in a cross-sectional European study. Eur Respir J.

[22] Jones PW, Harding G, Wiklund I, Berry P, Tabberer M, Yu R, Leidy NK (2012). Tests of the responsiveness of the COPD assessment test following acute exacerbation and pulmonary rehabilitation. Chest.

[23] Beyer-Westendorf J, Buller H (2011). External and internal validity of open label or double-blind trials in oral anticoagulation: better, worse or just different?. J Thromb Haemost.

[24] Beeh KM, Burgel PR, Franssen FM, Lopez-Campos JL, Loukides S, Hurst JR, Flezar M, Ulrik CS, Di Marco F, Stolz D, et al: How do dual long-acting bronchodilators prevent exacerbations of chronic obstructive pulmonary disease? Am J Respir Crit Care Med 2016. [Epub ahead of print]. 10.1164/rccm.201609-1794CI 27922741

[25] Kostikas K, Saifakas NM (2016). Does the term “deflators” reflect more accurately the beneficial effects of long-acting bronchodilators in COPD?. COPD.

[26] Beeh KM, Korn S, Beier J, Jadayel D, Henley M, D'Andrea P, Banerji D (2014). Effect of QVA149 on lung volumes and exercise tolerance in COPD patients: the BRIGHT study. Respir Med.

[27] Zhong N, Wang C, Zhou X, Zhang N, Humphries M, Wang L, Thach C, Patalano F, Banerji D (2015). LANTERN: a randomized study of QVA149 versus salmeterol/fluticasone combination in patients with COPD. Int J Chron Obstruct Pulmon Dis.

[28] Patalano F, Banerji D, D'Andrea P, Fogel R, Altman P, Colthorpe P (2014). Addressing unmet needs in the treatment of COPD. Eur Respir Rev.

