# Supplementary material for: Efficacy and safety of direct switch to indacaterol/glycopyrronium in patients with moderate COPD: the CRYSTAL open-label randomised trial
Source: Respir Res. 2017 Jul 18;18:140. doi: 10.1186/s12931-017-0622-x (PMC5516383; doi:10.1186/s12931-017-0622-x)
Supplement: Additional file 1: — Supplementary file for the CRYSTAL study. (DOCX 984 kb) [file 12931_2017_622_MOESM1_ESM.docx]

**Efficacy and safety of direct switch to indacaterol/glycopyrronium in patients with moderate COPD: The CRYSTAL open-label randomised trial**

- 1. **Overall design of the CRYSTAL study**

**Figure S1. Study design**

**
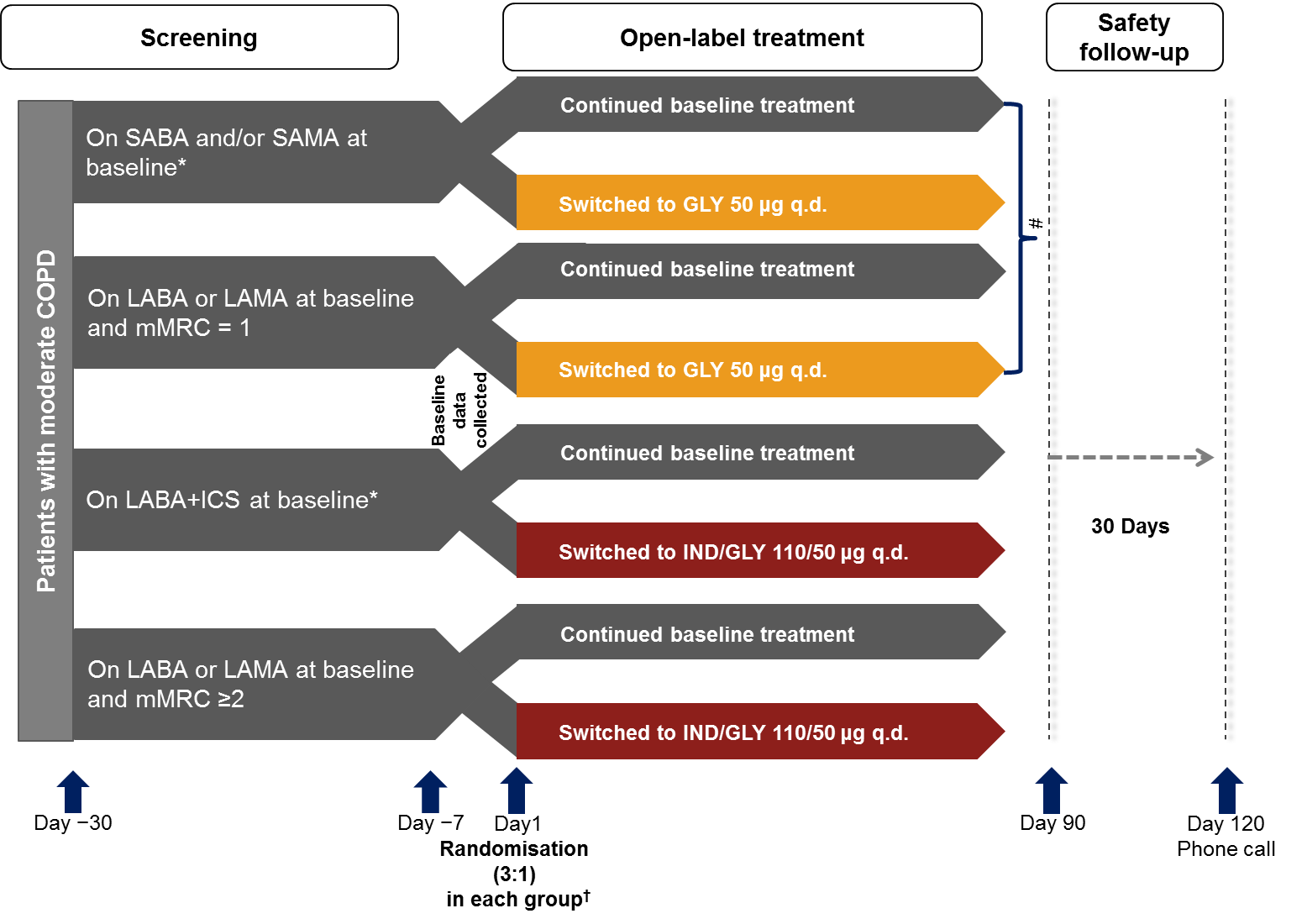
**

*Free or fixed-dose combination.

^†^Randomisation ratio (switched: baseline treatments) = 3:1 by stratifying background medications. ^#^Due to the low recruitment in GLY groups, inclusion of patients in these groups was stopped at the time that the randomization in IND/GLY groups was completed. All comparisons were for superiority of the switched treatment, with the exception of group 2, which was a non-inferiority analysis.

COPD, chronic obstructive pulmonary disease; GLY, glycopyrronium; ICS, inhaled corticosteroid; IND/GLY, indacaterol/glycopyrronium; LABA, long-acting β_2_-agonist; LAMA, long-acting muscarinic antagonist; mMRC, modified Medical Research Council; q.d., once daily; SABA, short-acting β_2_-agonist; SAMA, short-acting muscarinic antagonist

**S1.1. Inclusion criteria**

1. Patients who have signed an informed consent form before any assessment is performed
2. Men and women aged ≥40 years
3. Patients with moderate COPD according to the Global Initiative for Chronic Obstructive Lung Disease (GOLD) criteria, 2013
4. Current or ex-smokers who have a smoking history of at least 10 pack-years
5. Patients with airflow limitation indicated by a post-bronchodilator forced expiratory volume in 1 second (FEV_1_) ≥50% and <80% of the predicted normal value and a post-bronchodilator FEV_1_/forced vital capacity (FVC) <0.7 at Visit 2 (between Day -7 to Day 1)
6. Patients who have been on a stable dose of one of the following COPD baseline treatments for at least 3 months at Visit 1 (Day -30):
   1. Any short-acting β_2_-agonist (SABA) monotherapy (such as, but not limited to, salbutamol),
   2. Any short-acting muscarinic antagonist (SAMA) monotherapy (such as, but not limited to, ipratropium),
   3. Any SABA and SAMA in free or fixed-dose combination (FDC; such as, but not limited to, salbutamol/ipratropium),
   4. Any long-acting β_2_-agonist (LABA) monotherapy (such as, but not limited to, formoterol, salmeterol or indacaterol),
   5. Any long-acting muscarinic antagonist (LAMA) monotherapy (such as, but not limited to, tiotropium or aclidinium) except glycopyrronium bromide or
   6. Any LABA and inhaled corticosteroid (ICS) in free (such as, but not limited to, beclomethasone or fluticasone) or FDC (such as, but not limited to, salmeterol/fluticasone or formoterol/budesonide)
7. Patients with a modified Medical Research Council (mMRC) score ≥1 at Visit 1 (Day -30)

**S1.2. Exclusion criteria**

1. Patients with conditions contraindicated for treatment with or those with a history of reactions/hypersensitivity to any of the following inhalational drugs or to drugs of similar chemical classes or any component thereof: anti-cholinergic agents, long- and short-acting β_2_-adrenergic agonists, sympathomimetic amines, lactose or any other excipients of the trial medication
2. Patients with narrow-angle glaucoma, urinary retention or severe renal impairment (history of an estimated glomerular filtration rate [eGFR] <30 mL/min/1.73 m^2^ within 12 months before Visit 1 [Day -30]), including those with end-stage renal disease who require dialysis
3. Patients with an active/clinical history of asthma
4. A history of malignancy of any organ system (other than localised basal cell carcinoma of the skin), treated or untreated, within the past 5 years, regardless of an evidence of local recurrence or metastases
5. A documented history of >1 COPD exacerbations requiring treatment with systemic corticosteroids or antibiotics and/or hospitalisation in the past 12 months
   Patients who have not had a COPD exacerbation in the past 12 months or have developed a COPD exacerbation between screening (Visit 1 [Day -30]) and baseline (Visit 2 [Day -7]) will not be eligible but will be permitted to be re-screened after a minimum of 6 weeks after resolution of the COPD exacerbation
6. Patients who, in the judgment of the investigator, have a clinically relevant laboratory abnormality or a clinically significant condition such as (but not limited to) unstable ischaemic heart disease, left ventricular failure (New York Heart Association [NYHA] class III and IV), history of myocardial infarction, arrhythmia (excluding chronic stable atrial fibrillation), uncontrolled hypo- or hyperthyroidism, hypokalaemia or hyperadrenergic state or any condition that might compromise patient safety or compliance, interfere with evaluation or preclude study completion
7. A history of resting QTc (Fridericia preferred, but Bazett acceptable) >450 ms (men) or >460 ms (women) within 5 years before Visit 1 (Day -30)
8. Patients who are treated with glycopyrronium bromide at Visit 1 (Day -30)
   Patients on non-selective β-blockers; such patients may enter the study after withdrawal of the non-selective β-blocker during a 7-day washout period
9. Patients receiving any other prohibited COPD-related medications; prohibited COPD-related medications must undergo the required washout period before Visit 2 (Day -7)
10. Patients who are, in the opinion of the investigator, known to be unreliable or non-compliant
11. Patients with a body mass index (BMI) of >40 kg/m^2^
12. Use of other investigational drugs within 5 half-lives of enrolment or within 30 days, whichever is longer
13. Pregnant or nursing (lactating) women, where pregnancy is defined as the state of a woman after conception and until the termination of gestation, confirmed by a positive human chorionic gonadotropin (hCG) laboratory test
14. Women of childbearing potential, defined as all women who are physiologically capable of becoming pregnant, unless they are using effective contraception methods while being on the study treatment

**S1.3. Major protocol deviations**

Major protocol deviation (PD) is defined as any other PD that resulted in a significant risk to the patient’s safety. The reportable PDs were grouped into four different categories:

1. Patients who entered the study (thus included in the safety population) even though they did not satisfy the entry criteria (mainly patients who did not meet the inclusion criterion number 5 or number 6).
2. Patients who developed withdrawal criteria during the study but were not withdrawn (moderate or severe COPD exacerbations, use of other COPD treatment different from the study medication assigned at Visit 2 (Day -7) or interruption of investigational treatment for more than 5 consecutive days)
3. Patients who received the wrong treatment or incorrect dose
4. Patients who received an excluded concomitant treatment (mainly parenteral or oral corticosteroids)

**S1.4. Assessments**

The primary and secondary assessments were performed at baseline and Week 12:

1. Trough FEV_1_ was measured after the evening dose or the morning dose, depending on treatment.
2. Transition dyspnoea index (TDI) provides a measure of dyspnoea on a 17-unit scale ranging from +9 to −9; higher TDI scores indicate a greater improvement in dyspnoea.[1]
3. COPD Assessment Test (CAT) is an 8-item, self-administered, validated tool used to measure health status impairment in patients with COPD; the CAT score ranges between 0 and 40, and a score of ≥10 is an indicator of significantly impaired health status.[2]
4. COPD Clinical Questionnaire (CCQ) is a self-administered, 10-item questionnaire designed to measure COPD severity in clinical practice; lower CCQ scores represent greater improvement in health status.[3]
5. The mean number of puffs of rescue medication use and the percentage of days without rescue medication use were assessed using electronic patient diary (e-diary).

**S2. Disposition of patients in overall CRYSTAL study**

**Figure S2. Patient disposition**

**
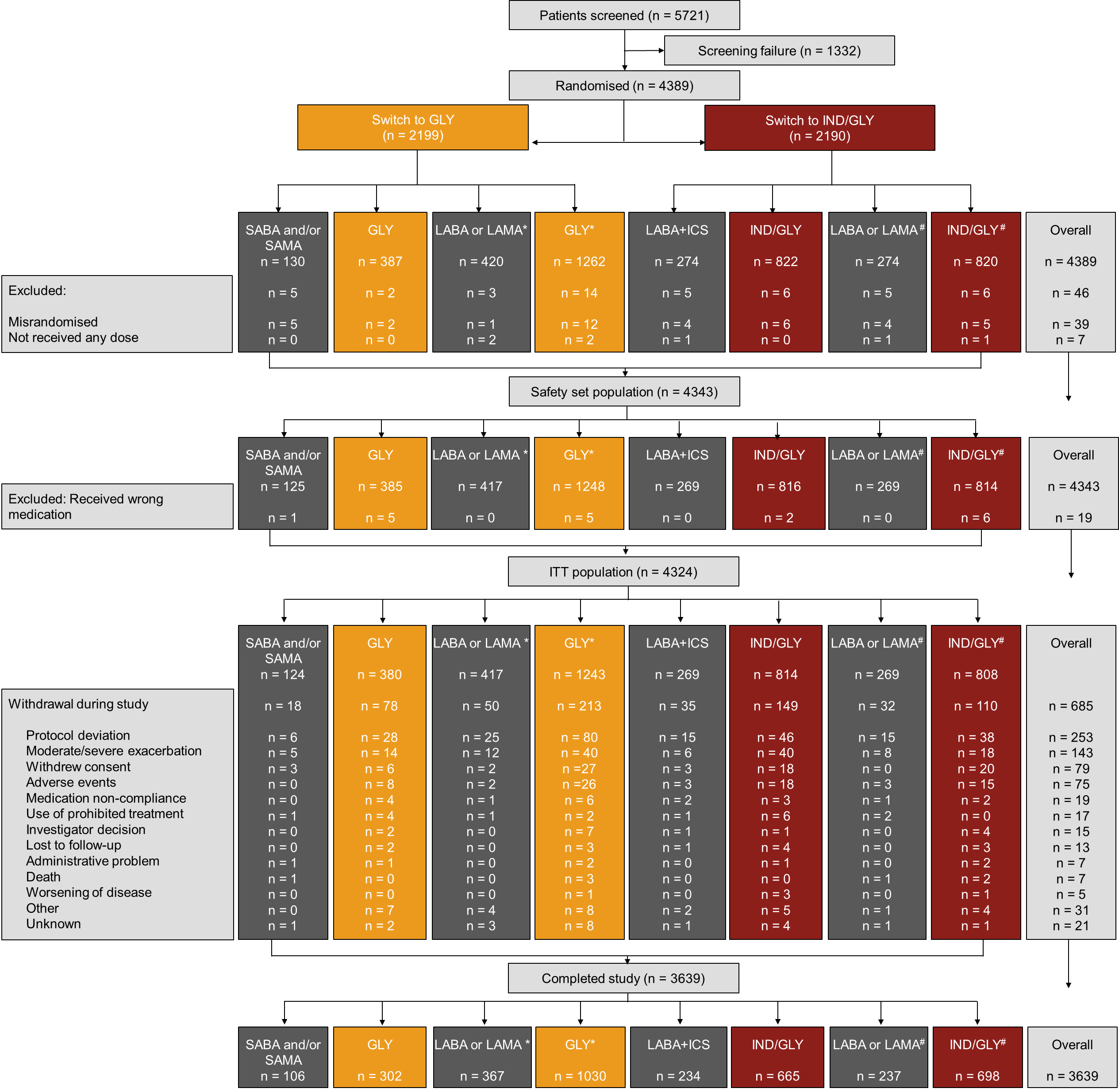
**

*Non-inferiority testing and patients had an mMRC score = 1; ^#^Patients had an mMRC score ≥2

Baseline characteristics of patients were recorded during the 7 days prior randomisation.

GLY, glycopyrronium; IND/GLY, indacaterol/glycopyrronium; ITT, intention to treat; LABA, long-acting β_2_-agonist; LAMA, long-acting muscarinic antagonist; LABA+ICS, long-acting β_2_-agonist+inhaled corticosteroid; mMRC, modified Medical Research Council; SABA, short-acting β_2_-agonist; SAMA, short-acting muscarinic antagonist

**S3. Subgroup analyses of responses to IND/GLY versus LABA+ICS or LABA or LAMA**

**Figure S3. Post-hoc subgroup analysis of improvement in trough FEV_1_ in various baseline characteristics with IND/GLY compared with (A) LABA+ICS and (B) LABA or LAMA**

| **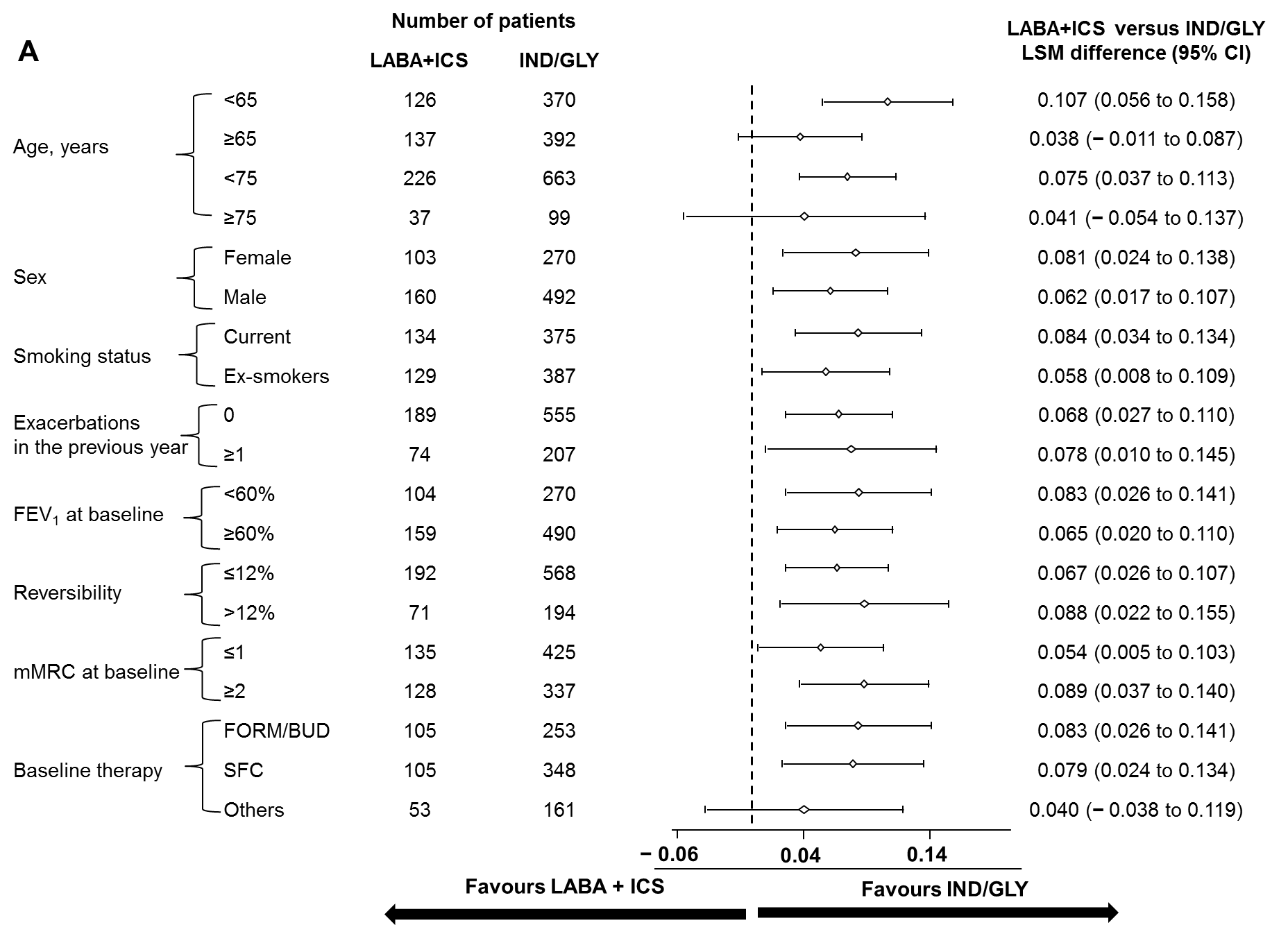**  **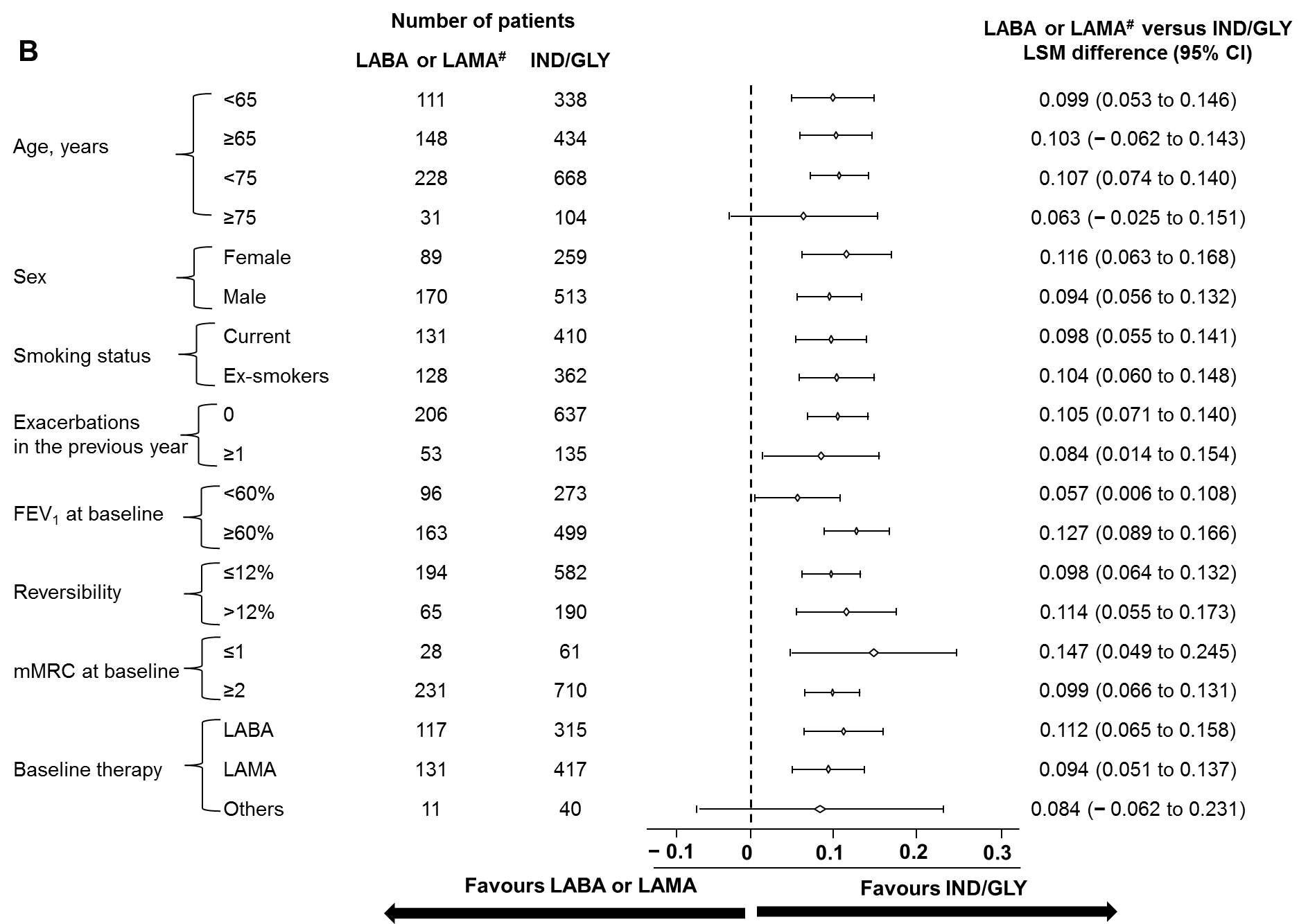** |
| --- |
| ^#^Patients had an mMRC score ≥2.  CI, confidence interval; FEV_1_, forced expiratory volume in 1 second; IND/GLY, indacaterol/glycopyrronium; LABA, long-acting β_2_-agonist; LAMA, long-acting muscarinic antagonist; LABA+ICS, long-acting β_2_-agonist+inhaled corticosteroid; LSM, least squares mean; mMRC, modified Medical Research Council; FORM/BUD, formoterol/budesonide fixed-dose combination; SFC, salmeterol/fluticasone fixed-dose combination |

**Figure S4. Post-hoc subgroup analysis of improvement in TDI total scores in various baseline characteristics with IND/GLY compared with (A) LABA+ICS and (B) LABA or LAMA**

| **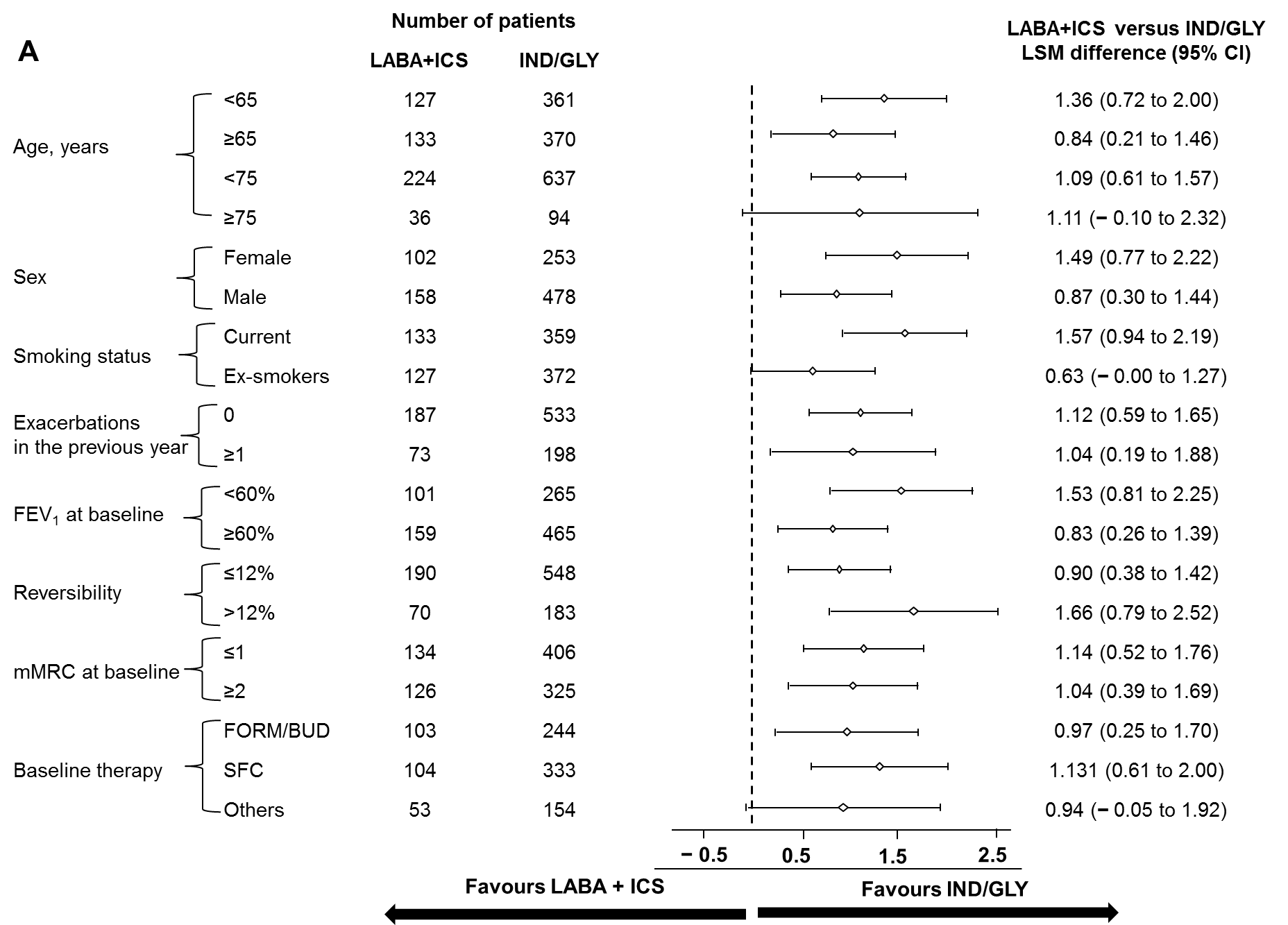**  **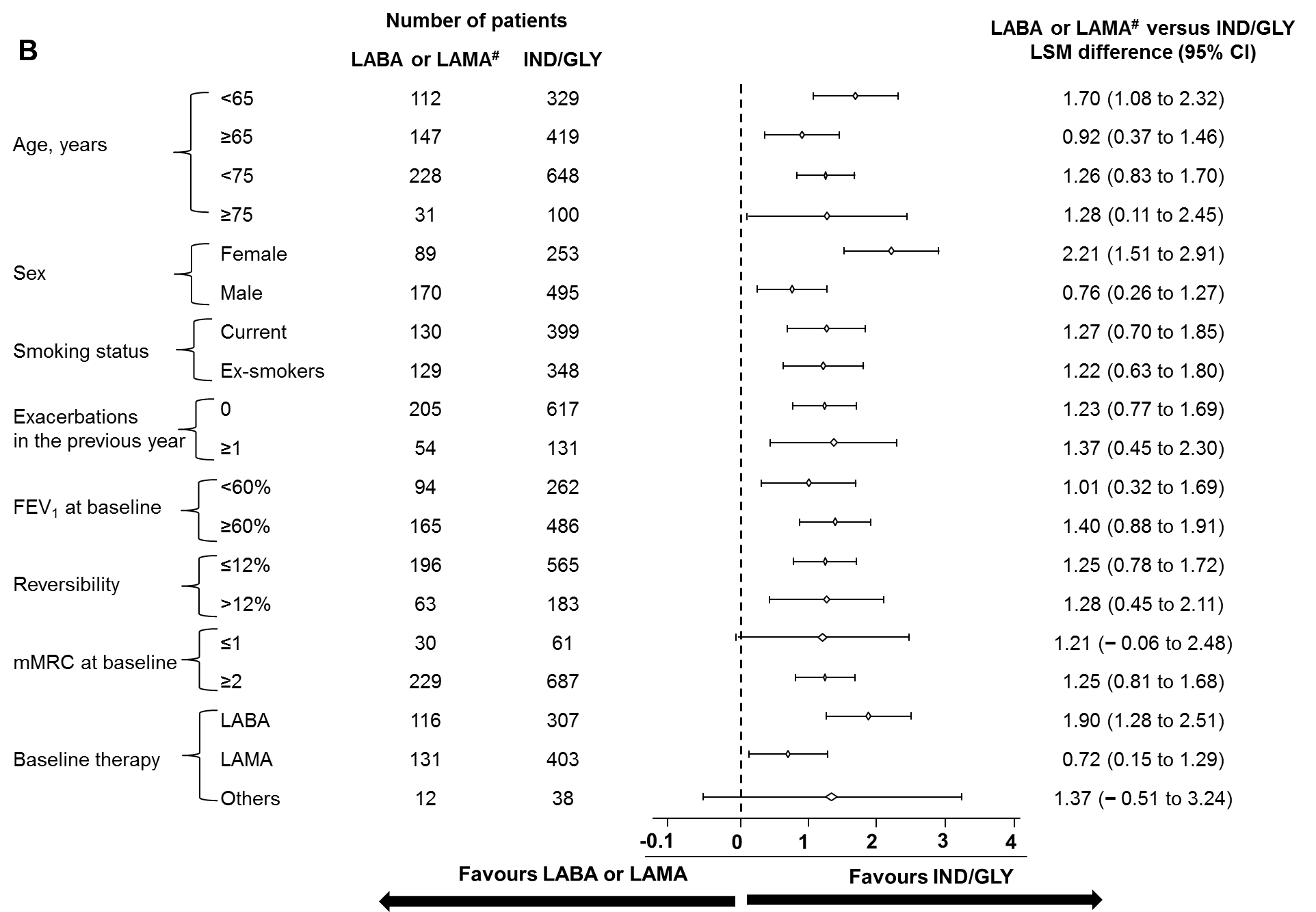** |
| --- |

^#^Patients had an mMRC score ≥2.

CI, confidence interval; FEV_1_, forced expiratory volume in 1 second; IND/GLY, indacaterol/glycopyrronium; LABA, long-acting β_2_-agonist; LAMA, long-acting muscarinic antagonist; LABA+ICS, long-acting β_2_-agonist+inhaled corticosteroid; LSM, least squares mean; mMRC, modified Medical Research Council; FORM/BUD, formoterol/budesonide fixed-dose combination; SFC, salmeterol/fluticasone fixed-dose combination; TDI, transition dyspnoea index

**S4. Statistical analysis of GLY groups**

The intention-to-treat (ITT) population consisted of all randomised patients who received at least one dose of the study medication, whereas the per-protocol (PP) population consisted of all patients in the ITT population with valid measurements of the primary endpoints without major PDs. Efficacy analyses were performed on both ITT and PP populations (primary endpoint only for the latter). Superiority testing for FEV_1_ and TDI at Week 12 (mean treatment differences [Δs] of ≥60 mL and ≥1 unit, respectively) was performed for glycopyrronium (GLY) versus SABA or SAMA as baseline therapy and indacaterol/glycopyrronium (IND/GLY) versus baseline therapy. In contrast, non-inferiority testing for FEV_1_ and TDI at 12 weeks (non-inferiority limits of 40 mL and 0.6 units, respectively) was performed for GLY versus LABA or LAMA as baseline therapy and an mMRC score of 1 point.

For the two co-primary endpoints (trough FEV_1_ and TDI after 12 weeks of treatment), a mixed model with treatment as a fixed effect and baseline value as a covariate was constructed.

Missing data imputation through last-observation-carried-forward (LOCF) approach for trough FEV_1_ and TDI total scores at Week 12 was performed for the ITT population when primary efficacy endpoints (trough FEV_1_ and/or TDI values) were collected at a visit immediately after a patient discontinued treatment.

Furthermore, the proportion of responders, i.e. patients reaching a minimal clinically significant change from baseline (100 mL in trough FEV_1_; 1 unit in TDI) at 12 weeks was presented and analysed using the observed odds ratio (OR) for responders versus non-responders. Secondary efficacy endpoints (CAT total scores, CCQ total scores, number of puffs of rescue medication and patient-reported symptoms of COPD) were analysed at Week 12 by using a non-parametric Mann–Whitney–Wilcoxon test. In addition, the proportion of CAT and CCQ responders (defined as a significant change from baseline of −2 and −0.4 units, respectively) were presented. All safety analyses were performed on the safety population, comprising all patients who received at least one dose of the study treatment.

IND/GLY groups achieved a study power of approximately 90%, whereas groups 1 and 2 were underpowered. The final power for GLY (switched from SABA and/or SAMA and LABA or LAMA) and ING/GLY (switched from LABA+ICS and LABA or LAMA) groups were 65%, 75%, 93% and 92%, respectively. Hence, statistical analysis for GLY groups may be inconclusive, and the results must be interpreted with caution. To show the superiority of GLY versus short-acting bronchodilators (SABA or SAMA) on trough FEV_1_, a two-group t-test with a 5% two-sided significance level would have 90% power to detect a difference in means of 60 mL, assuming that the common standard deviation was 250 mL, when the sample sizes in the two groups were 732 and 244, respectively (a total sample size of 976). Considering a drop out of about 5%, a total of 1,028 patients with SABA or SAMA as baseline treatment were needed to be randomised. To show the superiority of GLY versus short-acting bronchodilators (SABA or SAMA) on TDI, a two-group t-test with a 5% two-sided significance level would have 90% power to detect a difference in means of 1, assuming that the common standard deviation was 4, when the sample sizes in the two groups were 674 and 225, respectively (a total sample size of 899). Considering a drop out of about 5%, a total of 946 patients with SABA or SAMA as baseline treatment were needed to be randomised. To show the non-inferiority of GLY versus long-acting bronchodilators (LABA or LAMA monotherapy) on trough FEV_1_, a two-group t-test with a 2.5% one-sided significance level would have 90% power to detect a difference in means of 40 mL, assuming that the common standard deviation was 250 mL, when the sample sizes in the two groups were 1,644 and 548, respectively (a total sample size of 2,192). Considering a drop out of about 5%, a total of 2,308 patients with mMRC score = 1 point at Visit 1 (Day -30) and LABA or LAMA monotherapy as baseline treatment were needed to be randomised.

To show the non-inferiority of GLY versus long-acting bronchodilators (LABA or LAMA monotherapy) on TDI, a two-group t-test with a 2.5% one-sided significance level would have 90% power to detect a difference in means of 0.6, assuming that the common standard deviation was 4, when the sample sizes in the two groups were 1,870 and 624, respectively (a total sample size of 2,494). Considering a drop out of about 5%, a total of 2,628 patients with an mMRC score = 1 point at Visit 1 (Day -30) and LABA or LAMA monotherapy as baseline treatment were needed to be randomised. The co-primary hypotheses (within each stratum) were tested for both. Considering that both primary variables are needed to describe clinically-relevant treatment benefits, statistical significance is needed for both primary variables. Therefore, no formal alpha adjustment was necessary, thus alpha error was not requested (EMA CPMP/EWP/908/99).[4] However, this procedure inflates the relevant type II error, but inflation was taken into account changing the power from 80% to 90% also in the non-inferiority margin. Overall, i.e. considering the double testing, the power would at least be 80%.

**S5. Randomisation and treatment assignment in the CYRYSTAL study**

At Visit 2 (Day -7), all eligible patients were randomised via an Interactive Response Technology (IRT) to one of the eight random groups. The investigator or his/her delegate contacted the IRT after confirming that the patient fulfilled all the inclusion/exclusion criteria. The IRT assigned a randomisation number to the patient, which was used to link the patient to a random group and specified a unique medication number for the package of experimental treatment to be dispensed to the patient. The medication of the comparator had no unique medication number and was not assigned by the IRT. The randomisation numbers were generated using the following procedure to ensure that treatment assignment was unbiased and concealed from patients and investigator staff. A patient randomisation list was produced by the IRT provider using a validated system that automates the random assignment of patient numbers to randomisation numbers. These randomisation numbers were linked to the different random groups, which in turn were linked to medication numbers. A separate medication list was produced by or under the responsibility of Novartis DSM using a validated system that automates the random assignment of medication numbers to packs containing the investigational drug(s).

**S6. Results of GLY group**

**Table S1. Demographics and baseline characteristics of patients switched to GLY (ITT population)**

| **Characteristics** | **SABA and/or SAMA n = 122** | **GLY n = 369** | **LABA or LAMA^#^ n = 420** | **GLY^#^**  **n = 1254** |
| --- | --- | --- | --- | --- |
| Age, years | 64.1 (7.8) | 63.1 (8.4) | 64.6 (8.2) | 64.4 (8.2) |
| Gender – male, n (%) | 77 (63.1%) | 253 (68.6%) | 288 (68.6%) | 885 (70.6%) |
| Current smoker | 90 (73.8) | 220 (59.6) | 213 (50.7) | 669 (53.4) |
| Post-bronchodilator FEV_1_, L | 1.90 (0.5) | 1.90 (0.5) | 1.90 (0.4) | 1.91 (0.5) |
| Post-bronchodilator FEV_1_, % predicted of normal value | 66.5 (7.6) | 65.7 (8.7) | 66.1 (8.4) | 65.6 (8.7) |
| Dyspnoea – mMRC grade, n (%) |  |  |  |  |
| 0 | 0 (0.0%) | 7 (1.9%) | 1 (0.2%) | 13 (1.0%) |
| 1 | 89 (73.0%) | 260 (70.5%) | 404 (96.2%) | 1174 (93.6%) |
| ≥2 | 33 (27.1%) | 102 (27.6%) | 15 (3.6%) | 66 (5.3%) |
| Number of exacerbation in the past 12 months, n (%) |  |  |  |  |
| 0 | 99 (81.2%) | 300 (81.3%) | 341 (81.2%) | 1038 (82.8%) |
| 1 | 22 (18.0%) | 68 (18.4%) | 78 (18.6%) | 215 (17.2%) |
| ≥2 | 1 (0.8%) | 1 (0.3%) | 1 (0.2%) | 1 (0.1%) |
| Baseline treatments, n (%)^‡^ |  |  |  |  |
| LAMA (only monotherapy) | 1 (0.8%) | 3 (0.8%) | 236 (56.6%) | 687 (55.3%) |
| LABA (only monotherapy) | - | 2 (0.5%) | 176 (42.2%) | 546 (43.9%) |
| LABA+ICS (fixed-dose or free combination) | - | 1 (0.3%) | - | 3 (0.2%) |
| Others^†^ | 128 (103.2%) | 378 (99.5%) | 17 (4.0%) | 53 (4.3%) |
| ^#^Non-inferiority testing and patient had an mMRC score = 1; ^‡^At baseline, some of the patients were receiving more than one type of COPD medications. ^†^SABA, SAMA, ICS, systemic corticosteroids, methylxanthines, roflumilast, etc. Data are presented as mean (standard deviation), unless otherwise stated.  FEV_1_, forced expiratory volume in 1 second; GLY, glycopyrronium; ITT, intention to treat; LABA, long-acting β_2_-agonist; LAMA, long-acting muscarinic antagonist; mMRC, modified Medical Research Council; SABA, short-acting β_2_-agonist; SAMA, short-acting muscarinic antagonist | | | | |

## **S6.1. Impact of direct switch to GLY from baseline treatments on lung function and dyspnoea**

In the early discontinued groups of direct switch to GLY from SABA and/or SAMA and LABA or LAMA with an mMRC score = 1, which were underpowered due to a small sample size, GLY provided superior improvements in trough FEV_1_ (Δ = +65 mL, P = 0.018) and TDI (Δ = 1.79 units, P < 0.0001) at Week 12 against SABA and/or SAMA (**Figures S5A, S5B**).

In addition, GLY showed non-inferiority in terms of achieving Δ as a lower bound of the 95% confidence interval (CI) of −40 mL for FEV_1_ and the lower bound of the 95% CI of 0.6 units for TDI versus LABA or LAMA at Week 12 (**Figures S6A and S6B**).

Furthermore, a significantly greater proportion of patients reached the minimum clinically important difference (MCID) of 100 mL for trough FEV_1_ and ≥1 unit for TDI compared with SABA and/or SAMA and LABA or LAMA at Week 12 (**Table S2**).

**S6.2. Impact of direct switch to GLY on patient-reported outcomes and safety**

In the ITT population, patients who received GLY experienced greater reductions in total CAT scores, CCQ total scores and number of puffs of rescue medication per day and increase in days without rescue medication use versus those who received SABA and/or SAMA (**Table S3**). Four deaths were reported in the GLY group during the study, and none were considered to be treatment related: one death was reported in the group that remained on SABA and/or SAMA and three in the group that switched to GLY from LABA or LAMA. Moreover, the safety and tolerability of GLY was consistent with previous reports without any new safety signals (**Tables S4 and S5**).

**Figure S5. Change from baseline in (A) trough FEV_1_ and (B) TDI with GLY treatment versus other treatments at Week 12 (ITT population)**

| **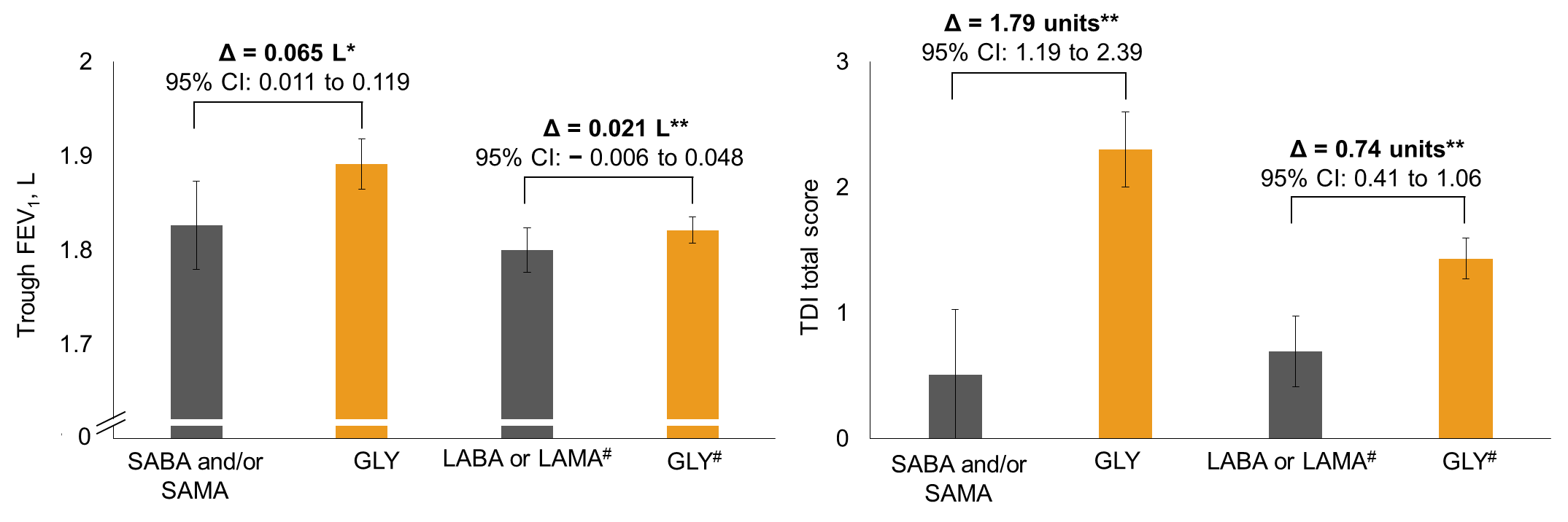** |
| --- |
| *P = 0.0180; **P < 0.0001; ^#^Non-inferiority testing and patient had an mMRC score = 1; Data are least squares means (95% CI).  Δ, treatment difference; CI, confidence interval; FEV_1_, forced expiratory volume in 1 second; GLY, glycopyrronium; ITT, intention to treat; LABA, long-acting β_2_-agonist; LAMA, long-acting muscarinic antagonist; mMRC, modified Medical Research Council; SABA, short-acting β_2_-agonist; SAMA, short-acting muscarinic antagonist, TDI, transition dyspnoea index |

**Figure S6. Change from baseline in (A) trough FEV_1_ and (B) dyspnoea (TDI) with GLY compared with LABDs (non-inferiority analysis) at Week 12 (ITT population)**

| **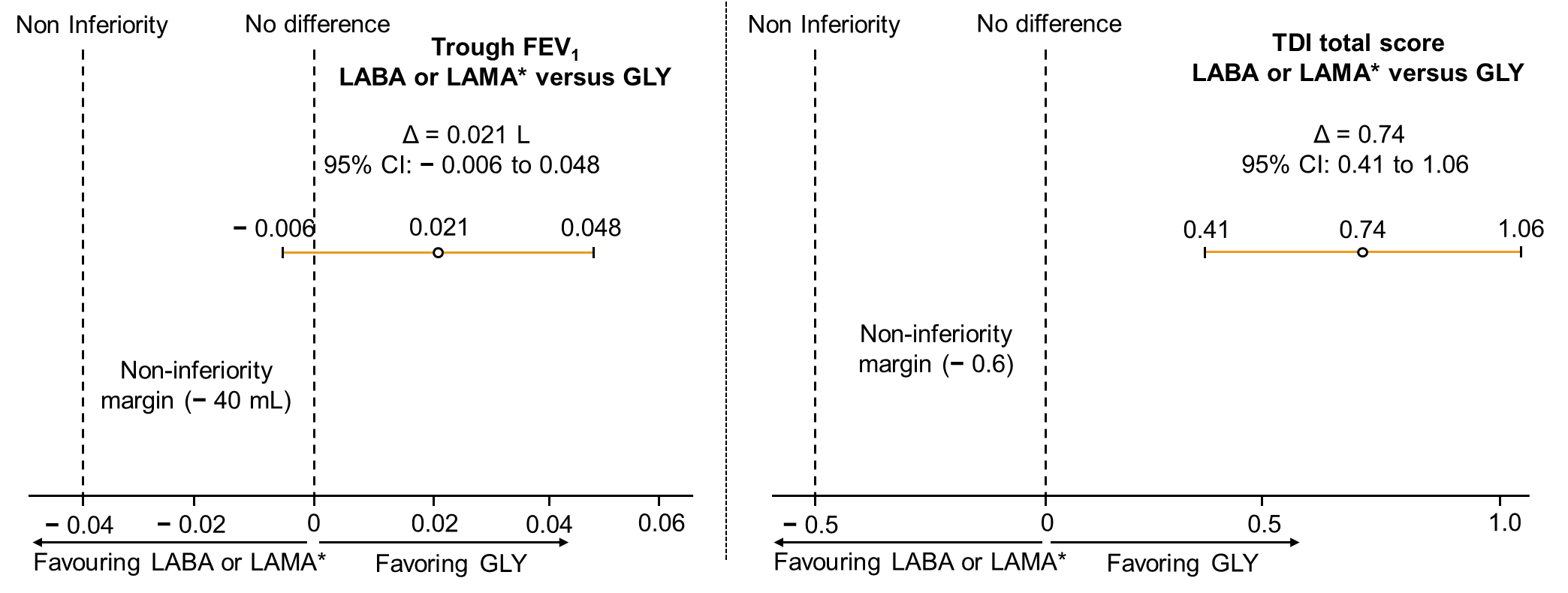** |
| --- |
| ^*^Non-inferiority testing and patient had an mMRC score = 1; Data are least squares means (95% CI); Δ, treatment difference; CI, confidence interval; FEV_1_, forced expiratory volume in 1 second; GLY, glycopyrronium; ITT, intention to treat; LABA, long-acting β_2_-agonist; LABDs, long-acting bronchodilators; LAMA, long-acting muscarinic antagonist; mMRC, modified Medical Research Council; SABA, short-acting β_2_-agonist; SAMA, short-acting muscarinic antagonist; TDI, transition dyspnoea index |

**Table S2. Effects of direct switch to GLY from baseline treatments on trough FEV_1_ and TDI total scores: proportion of patients achieving MCID (ITT population)**

|  | **SABA and/or SAMA**  **n = 122** | **GLY**  **n = 369** | **LABA or LAMA^#^**  **n = 420** | **GLY^#^**  **n = 1254** |
| --- | --- | --- | --- | --- |
| Patients achieved MCID in trough FEV_1_ (a ≥100-mL difference from baseline) | 44 (36.1%) | 181 (49.1%) | 111 (26.4%) | 413 (32.9%) |
| Trough FEV_1_ responders (OR [95% CI]) | 1.77 (1.15 to 2.72) | | 1.40 (1.09 to 1.80) | |
| Patients achieved MCID in TDI total score (a ≥1-unit difference from baseline) | 30 (24.6%) | 212 (57.5%) | 110 (26.2%) | 577 (46.0%) |
| TDI responders (OR [95% CI]) | 4.58 (2.86 to 7.34) | | 2.57 (2.00 to 3.30) | |
| ^#^Non-inferiority testing and patient had an mMRC score = 1. Data are presented as number of incidents (%), unless otherwise stated.  CI, confidence interval; FEV_1_, forced expiratory volume in 1 second; GLY, glycopyrronium; ITT, intention to treat; LABA, long-acting β_2_-agonist; LAMA, long-acting muscarinic antagonist; MCID, minimum clinically important difference; mMRC, modified Medical Research Council; OR, odds ratio; SABA, short-acting β_2_-agonist; SAMA, short-acting muscarinic antagonist; TDI, transition dyspnoea index | | | | |

**Table S3. Effects of a direct switch to GLY from baseline treatments on CAT, CCQ and rescue medication use (ITT population)**

|  | **SABA and/or SAMA n = 122** | | **GLY n = 369** | **LABA or LAMA^#^ n = 420** | | **GLY^#^**  **n = 1254** |
| --- | --- | --- | --- | --- | --- | --- |
| Total CAT score, change from baseline at Week 12 | 0.1 (4.6) | | −1.8 (5.3)*** | 0.1 (4.9) | | −0.5 (4.6) |
| Patients who achieved MCID in total CAT score (a ≥2-units difference from baseline), n (%) | 38 (31.2%) | | 155 (42.0%) | 129 (30.7%) | | 400 (31.90%) |
| CAT responders (a ≥2-unit decrease)^†^ | 1.93 (1.22 to 3.04) | | | 1.18 (0.92 to 1.51) | | |
| Total CCQ score, change from baseline at Week 12 | −0.0 (0.6) | −0.3 (0.7)*** | | 0.0 (0.7) | −0.1 (0.7)** | |
| Patients who achieved MCID in total CCQ score (a ≥0.4-unit difference from baseline), n (%) | 22 (18.0%) | 105 (28.5%) | | 81 (19.3%) | 291 (23.2%) | |
| CCQ responders (a ≥0.4-unit decrease)^†^ | 2.04 (1.20 to 3.45) | | | 1.40 (1.06 to 1.86) | | |
| Number of puffs of rescue medication over 12 weeks | 1.8 (1.7) | 1.0 (1.3)**** | | 0.8 (1.2) | 0.7 (1.1) | |
| Percentage of days without rescue medication use over 12 weeks | 32.8 (41.9) | 55.1 (42.6)**** | | 60.4 (42.3) | 58.3 (42.7) | |
| *P < 0.05; **P < 0.01; ***P < 0.001; ****P < 0.0001. ^#^Non-inferiority testing and patient had an mMRC score = 1. ^†^Odds ratios are displayed (95% CI) and analysed in the ITT population. Data are presented as mean (standard deviation), unless otherwise specified.  CI, confidence interval; CAT, COPD Assessment Test; CCQ, COPD Clinical Questionnaire; GLY, glycopyrronium; ITT, intention to treat; LABA, long-acting β_2_-agonist; LAMA, long-acting muscarinic antagonist; MCID, minimal clinically important difference; mMRC, modified Medical Research Council; SABA, short-acting β_2_-agonist; SAMA, short-acting muscarinic antagonist | | | | | | |

**Table S4. Treatment-emergent adverse events and serious adverse events during the study period (safety set)**

|  | **SABA and/or SAMA n = 125** | **GLY n = 385** | **LABA or LAMA^#^ n = 417** | **GLY* n = 1248** |
| --- | --- | --- | --- | --- |
| Any adverse event | 38 (30.4%) | 122 (31.7%) | 104 (24.9%) | 316 (25.3%) |
| Any serious adverse event | 4 (3.2%) | 9 (2.3%) | 11 (2.6%) | 30 (2.4%) |
| Any suspected drug-related adverse event | 0 (0.0%) | 15 (3.9%) | 3 (0.7%) | 38 (3.0%) |
| Any suspected drug-related serious adverse event | 0 (0.0%) | 0 (0.0%) | 0 (0.0%) | 2 (0.2%) |
| Any adverse event leading to treatment withdrawal | 0 | 12 (3.1%) | 3 (0.7%) | 29 (2.3%) |
| Any adverse event with fatal outcome (deaths) | 1 (0.8%) | 0 (0.0%) | 0 (0.0%) | 3 (0.2%) |
| ^#^Non-inferiority testing and patient had an mMRC score = 1. Data are presented as number of incidents (%).  GLY, glycopyrronium; LABA, long-acting β_2_-agonist; LAMA, long-acting muscarinic antagonist; mMRC, modified Medical Research Council; SABA, short-acting β_2_-agonist; SAMA, short-acting muscarinic antagonist | | | | |

**S6.3. Conclusions of results obtained from GLY groups**

In the study groups with a direct switch to GLY from previous treatments (**Figure S1**), all primary endpoints were met. GLY showed superiority in improving trough FEV_1_ and TDI total scores versus SABA and/or SAMAs and non-inferiority versus previous LABA or LAMA. The efficacy and safety of GLY were evaluated through several clinical trials in the GLOW programme, which revealed comparable improvements in lung function (trough FEV_1_), dyspnoea (TDI total score), health status (reduction in St. George’s Respiratory Questionnaire total score) and rescue medication use compared with tiotropium in patients with moderate COPD.[5, 6] Although no explanatory trials were conducted for direct comparison between GLY and a LABA, the SHINE study reported comparable efficacy and safety of GLY and IND.[7] Although these results of a direct switch to GLY in a clinical practice setting are consistent with the outcomes of previous randomised controlled trials (RCTs), these must be interpreted with caution due to the early stopping of GLY groups, which, consequently, was underpowered.

**S7. Overall adverse events in the CRYSTAL studySection**

**Table S5. Serious adverse events during the study period in all groups (safety set)**

|  | **SABA and/or SAMA n = 125** | **GLY n = 385** | **LABA or LAMA* n = 417** | **GLY* n =1248** | **LABA+ICS n = 269** | **IND/GLY n = 816** | **LABA or LAMA^#^ n = 269** | **IND/GLY n = 814^#^** |
| --- | --- | --- | --- | --- | --- | --- | --- | --- |
| **Number of patients with any treatment-emergent SAE** | 4 (3.2%) | 9 (2.3%) | 11 (2.6%) | 30 (2.4%) | 6 (2.2%) | 22 (2.7%) | 10 (3.7%) | 34 (4.2%) |
| **Number of treatment-emergent SAEs** | 6 | 15 | 12 | 43 | 8 | 24 | 14 | 49 |
| **System organ class** Preferred term |  |  |  |  |  |  |  |  |
| **Cardiac disorders** | 2 (1.6%) | 2 (0.5%) | 1 (0.2%) | 6 (0.5%) | 1 (0.4%) | 2 (0.3%) | 3 (1.1%) | 5 (0.6%) |
| Myocardial infarction | 1 (0.8%) | - | - | 1 (0.1%) | - | - | 1 (0.4%) | 1 (0.1%) |
| Atrial fibrillation | - | 1 (0.3%) | - | 1 (0.1%) | - | 1 (0.1%) | - | - |
| Coronary artery disease | - | 2 (0.5%) | 1 (0.2%) | - | - | - | - | - |
| Acute myocardial infarction | - | - | - | 1 (0.1%) | - | - | - | 1 (0.1%) |
| Myocardial ischaemia | - | - | - | 1 (0.1%) | - | - | 1 (0.4%) | - |
| Acute coronary syndrome | - | - | - | 1 (0.1%) | - | - | - | - |
| Angina pectoris | - | - | - | - | - | - | - | 1 (0.1%) |
| Angina unstable | - | - | - | - | - | - | 1 (0.4%) | - |
| Arrhythmia | - | - | - | 1 (0.1%) | - | - | - | - |
| Arteriosclerosis coronary artery | - | - | - | - | 1 (0.4%) | - | - | - |
| Cardiac arrest | 1 (0.8%) | - | - | - | - | - | - | - |
| Cardiac failure | - | - | - | - | - | - | - | 1 (0.1%) |
| Left ventricular failure | - | - | - | - | - | - | - | 1 (0.1%) |
| Supraventricular tachycardia | - | - | - | - | - | 1 (0.1%) | - | - |
| Tachycardia | - | 1 (0.3%) | - | - | - | - | - | - |
| **Infections and infestations** | - | 1 (0.3%) | 2 (0.5%) | 2 (0.2%) | 1 (0.4%) | 1 (0.1%) | 1 (0.4%) | 10 (1.2%) |
| Pneumonia | - | 1 (0.3%) | - | 2 (0.2%) | - | 1 (0.1%) | 1 (0.4%) | 7 (0.9%) |
| Bronchitis | - | - | 1 (0.2%) | - | - | - | - | 1 (0.1%) |
| Cystitis | - | - | - | - | 1 (0.4%) | - | - | - |
| Diverticulitis | - | - | 1 (0.2%) | - | - | - | - | - |
| Influenza | - | - | - | - | - | - | - | 1 (0.1%) |
| Mediastinal abscess | - | - | - | - | - | - | - | 1 (0.1%) |
| Oesophageal candidiasis | - | - | - | - | - | - | - | 1 (0.1%) |
| **Respiratory, thoracic and mediastinal disorders** | 1 (0.8%) | 1 (0.3%) | 2 (0.5%) | 4 (0.3%) | 1 (0.4%) | 4 (0.5%) | 2 (0.7%) | 4 (0.5%) |
| Chronic obstructive pulmonary disease | - | 1 (0.3%) | 1 (0.2%) | 3 (0.2%) | 1 (0.4%) | 3 (0.4%) | 1 (0.4%) | 3 (0.4%) |
| Pulmonary embolism | - | - | 1 (0.2%) | - | - | - | 1 (0.4%) | 1 (0.1%) |
| Pneumothorax | - | - | - | - | - | - | - | 1 (0.1%) |
| Pulmonary haemorrhage | 1 (0.8%) | - | - | - | - | - | - | - |
| Respiratory depression | - | - | - | - | - | 1 (0.1%) | - | - |
| Any TEAEs with fatal outcome (deaths) | 1 (0.8%) |  |  | 3 (0.2%) |  |  | 2 (0.7%) | 2 (0.3%) |
| *Patients had an mMRC score = 1; ^#^Patients had an mMRC score ≥ 2.  Data are presented as number of incidences (%)  GLY, glycopyrronium; IND/GLY, indacaterol/glycopyrronium; LABA, long-acting β_2_-agonist; LAMA, long-acting muscarinic antagonist; LABA+ICS, long-acting β_2_-agonist+inhaled corticosteroid; mMRC, modified Medical Research Council; SABA, short-acting β_2_-agonist; SAE, serious adverse event; SAMA, short-acting muscarinic antagonist; TEAEs, treatment-emergent adverse events | | | | | | | | |

**References:**

1. Mahler DA, Waterman LA, Ward J, McCusker C, ZuWallack R, Baird JC: **Validity and responsiveness of the self-administered computerized versions of the baseline and transition dyspnea indexes.** *Chest* 2007, **132:**1283-1290.

2. Gupta N, Pinto LM, Morogan A, Bourbeau J: **The COPD assessment test: a systematic review.** *Eur Respir J* 2014, **44:**873-884.

3. Kon SS, Dilaver D, Mittal M, Nolan CM, Clark AL, Canavan JL, Jones SE, Polkey MI, Man WD: **The Clinical COPD Questionnaire: response to pulmonary rehabilitation and minimal clinically important difference.** *Thorax* 2014, **69:**793-798.

4. **Points to consider on municipality issues in clinical trials. Committee for proprietary medicinal products (CPMP). CPMP/EWP/908/99.** *The European agency for the evaluation of medicinal products Evaluation of medicines for human use* 2002**:**London. September 19.

5. Chapman KR, Beeh KM, Beier J, Bateman ED, D'Urzo A, Nutbrown R, Henley M, Chen H, Overend T, D'Andrea P: **A blinded evaluation of the efficacy and safety of glycopyrronium, a once-daily long-acting muscarinic antagonist, versus tiotropium, in patients with COPD: the GLOW5 study.** *BMC Pulm Med* 2014, **14:**4.

6. Kerwin E, Hebert J, Gallagher N, Martin C, Overend T, Alagappan VK, Lu Y, Banerji D: **Efficacy and safety of NVA237 versus placebo and tiotropium in patients with COPD: the GLOW2 study.** *Eur Respir J* 2012, **40:**1106-1114.

7. Vogelmeier CF, Bateman ED, Pallante J, Alagappan VK, D'Andrea P, Chen H, Banerji D: **Efficacy and safety of once-daily QVA149 compared with twice-daily salmeterol-fluticasone in patients with chronic obstructive pulmonary disease (ILLUMINATE): a randomised, double-blind, parallel group study.** *Lancet Respir Med* 2013, **1:**51-60.

**S8. List of Principal Investigators of the CRYSTAL study:**

- Bernhard Forstner, Austria
- Dorninger Hans-Peter, Austria
- Flicker Martin, Austria
- Gerhard Köberl, Austria
- Grillenberger Johann, Austria
- Huber Alexander, Austria
- Josef Würtz, Austria
- Mahmud Sweilem, Austria
- Messner Jose, Austria
- Asandei Raluca, Belgium
- Baldassarre Sandra, Belgium
- Bauler Alain, Belgium
- Beutels Michel, Belgium
- Bolly Antoine, Belgium
- Bomans Peter, Belgium
- Butenda Dominique, Belgium
- Cataldo Didier, Belgium
- Compere Christophe, Belgium
- Corhay Jean-Louis, Belgium
- Daniël Vantroyen, Belgium
- De Fooz Geoffroy, Belgium
- De Munck Luc, Belgium
- De Saedeleer Bart, Belgium
- Delobbe Alain, Belgium
- Derijcke Miguel, Belgium
- Eykerman Tom, Belgium
- Fievet Frederic, Belgium
- Fremault Antoine, Belgium
- Guy Vereecken, Belgium
- Jan Behets, Belgium
- Liistro Giuseppe, Belgium
- Lins Muriel, Belgium
- Luc Van Zandweghe, Belgium
- Malysse Ines, Belgium
- Marc De Meulemeester, Belgium
- Martinot Jean-Benoit, Belgium
- Palem Alain, Belgium
- Rafaël Verlinden, Belgium
- Remels Linda, Belgium
- Sadowska Anna, Belgium
- Stéphane Vandenbemden, Belgium
- Vanmaele Luc, Belgium
- Yohan Balthazar, Belgium
- Yvan Calozet, Belgium
- Ali Masroor, Czech Republic
- Bártů Václava, Czech Republic
- Brejchová Martina, Czech Republic
- Burešová Marie, Czech Republic
- Bursová Jana, Czech Republic
- Chladil Petr, Czech Republic
- Čierná-Peterová Ivana, Czech Republic
- Čmakalová Martina, Czech Republic
- Davidová Romana, Czech Republic
- Dindoš Ján, Czech Republic
- Eliasová Blanka, Czech Republic
- Filipová Petra, Czech Republic
- Hadrava Martin, Czech Republic
- Hanák Vladimír, Czech Republic
- Holub Stanislav, Czech Republic
- Kašák Viktor, Czech Republic
- Kociánová Jana, Czech Republic
- Kolaříková Renata, Czech Republic
- Krejbich František, Czech Republic
- Křepelka Jan, Czech Republic
- Křížová Eva, Czech Republic
- Mechlová Charlotta, Czech Republic
- Pavlišová Ilona, Czech Republic
- Presperinová Jolana, Czech Republic
- Quadrátová Eva, Czech Republic
- Sklenář Milan, Czech Republic
- Veverka Josef, Czech Republic
- Woznicová Alexandra, Czech Republic
- Zitková Miluše, Czech Republic
- Houmann-Hammer Jacob, Denmark
- Iuel Gunvor, Denmark
- Sørensen Torben, Denmark
- Torp Claus Rydahl, Denmark
- Vinberg Jørgen, Denmark
- Jõgi Rain, Estonia
- Kirs Üllar, Estonia
- Meren Mari, Estonia
- Samarüütel Priit, Estonia
- Sreitor Zanna, Estonia
- Täht Ingrid, Estonia
- Bernady Alain, France
- Boumedienne Bendehiba, France
- Boyer Guy-René, France
- Cantin Thierry, France
- Claussner Martine, France
- Ducolone Alain, France
- Guilleminault Laurent, France
- Herer Bertrand, France
- Larrousse Mathieu, France
- Legendre Marc, France
- Marchand-Adam Sylvain, France
- Marty Jacques, France
- Meziane Lahouari, France
- Pegliasco Hervé, France
- Pigearias Bernard, France
- Tardif Philippe, France
- Thomas Similowski, France
- Verbert Antoine, France
- Xanthopoulos Athanasios, Germany
- Abts Markus, Germany
- Askar Bader, Germany
- Balck Katharina, Germany
- Ballenberger Sabine, Germany
- Balzer Roland, Germany
- Bäumer Anselm, Germany
- Beck Ekkehard, Germany
- Beckmann Bernhard, Germany
- Benduhn Henning, Germany
- Benedix Andreas, Germany
- Berger-Roscher Jürgen, Germany
- Bergtholdt Bettina, Germany
- Bessler Hartmut, Germany
- Bettig Ute, Germany
- Bewig Burkhard, Germany
- Bollmann Lutz, Germany
- Born Thorsten, Germany
- Botzen Ulrich, Germany
- Bourgeois Gerd, Germany
- Breyer Gerhard, Germany
- Brüggen Hans, Germany
- Brust Daniel, Germany
- Budweiser Stephan, Germany
- Chevts Julia, Germany
- Contzen Christel, Germany
- Corvinus Christoph, Germany
- de Roux Andrés, Germany
- Deckelmann Regina, Germany
- Deimling Andreas, Germany
- Eckhard Johannes-Georg, Germany
- Eckhardt Gerald, Germany
- Einenkel Andreas, Germany
- Eisel Erika, Germany
- Eisenschmidt Sven, Germany
- Eißfeller Enno, Germany
- Eissing Volker, Germany
- Engelhard Ralf, Germany
- Esmandar Mohamed, Germany
- Feldmeyer Frank, Germany
- Feussner Wolfram, Germany
- Foerster Karin, Germany
- Franke Christian, Germany
- Franz Karl-Heinz, Germany
- Fritzsche Andreas, Germany
- Fuchs Florian, Germany
- Gams Werner, Germany
- Gehling Umberto, Germany
- Gerbaulet Uwe, Germany
- Germann Hans, Germany
- Gernhold Markus, Germany
- Geßner Christian, Germany
- Ginko Thomas, Germany
- Graf Hans-Josef, Germany
- Granops Helge, Germany
- Grigat Christine, Germany
- Grimm-Sachs Vera, Germany
- Groth Gesine, Germany
- Haase Peter Uwe, Germany
- Hagemann Dirk, Germany
- Hagenow Andreas, Germany
- Hamm Hinrich, Germany
- Hammerl Peter, Germany
- Hampf Jürgen, Germany
- Hartung Hans-Christoph, Germany
- Heindl Stefan, Germany
- Heinze Alexander, Germany
- Hennig Rudolph, Germany
- Herfort Peter, Germany
- Hergdt Gunter, Germany
- Herold Jürgen, Germany
- Herrmann Petra, Germany
- Heymer Peter, Germany
- Hoefer Markus, Germany
- Hoffmann Martin, Germany
- Hoheisel Gerhard, Germany
- Hüting Reinhard, Germany
- Illies Gabriele, Germany
- Jaeger Burkhard, Germany
- Jandl Margret, Germany
- Jansen Volker, Germany
- Janssen Edgar, Germany
- Jezek Michael, Germany
- John Matthias, Germany
- Jung Thomas, Germany
- Junggeburth Josef, Germany
- Kaa Armin, Germany
- Kaiser Anatoli, Germany
- Kampschulte Hans-Jörg, Germany
- Kanniess Frank, Germany
- Kässner Frank, Germany
- Kehm Stefan, Germany
- Keller Claus, Germany
- Kemmerich Bernd, Germany
- Khariouzov Andrej, Germany
- Kleinecke-Pohl Uwe, Germany
- Klünsch Hans-Lothar, Germany
- Knie Alexandra, Germany
- Köchel Annemone, Germany
- Korduan Margit, Germany
- Kösters Frank, Germany
- Kroegel Claus, Germany
- Kroell Monika, Germany
- Kroenig Matthias, Germany
- Kroker Axel, Germany
- Kropp Maximilian, Germany
- Krüll Matthias, Germany
- Kuehne Petra, Germany
- Landers Bernhard, Germany
- Laßmann Hendrik, Germany
- Lehmann Andrei, Germany
- Lenk Ute, Germany
- Liedtke Michael, Germany
- Lienert Thomas, Germany
- Linnhoff Anneliese, Germany
- Luttermann Matthias, Germany
- Marten Irmgard, Germany
- Maus Olga, Germany
- Meixner Marcus, Germany
- Menke Thomas, Germany
- Mikloweit Petra, Germany
- Mozzilli Angelo, Germany
- Müller Hilger, Germany
- Nischik Ruth, Germany
- Oehlschläger Günther, Germany
- Oelker Jürgen, Germany
- Overlack Axel, Germany
- Peldschus Meike, Germany
- Pfeuffer Hans-Peter, Germany
- Piechatzek Richard, Germany
- Pitule Hannelore, Germany
- Plaßmann Georg, Germany
- Polke Klaus, Germany
- Rau Thorsten, Germany
- Redlich Ronald, Germany
- Riegel Antje, Germany
- Safavi Katayoon, Germany
- Sauer Rüdiger, Germany
- Schaper Lennart, Germany
- Schaper Martin, Germany
- Scheer Mathias, Germany
- Schenkenberger Isabelle, Germany
- Schiffer Clemens, Germany
- Schilder Cordula, Germany
- Schlegel Volker, Germany
- Schlenska Christian, Germany
- Schmidt Anne-Kathrin, Germany
- Schmidtmann Sören, Germany
- Schmitt Harald, Germany
- Schneider Thomas, Germany
- Schuermann Wolfgang, Germany
- Schulz Martin, Germany
- Schulze Jörg, Germany
- Schwab Tatjana, Germany
- Sebert Michael, Germany
- Seese Bernd, Germany
- Seiz Volker, Germany
- Sommer Claudia, Germany
- Sommerwerck Urte, Germany
- Sorichter Stephan, Germany
- Spengler Kai-Peter, Germany
- Sperling Karsten, Germany
- Spreda Frank, Germany
- Steffen Heiner, Germany
- Steinebach Inga, Germany
- Steinhauser Ulrich, Germany
- Stolpe Christoph, Germany
- Sudhoff Harald, Germany
- Sutor Gerd-Christian, Germany
- Taraben Abdo, Germany
- Trauth Herrmann A, Germany
- Tyler Kerstin, Germany
- Uebel Peter, Germany
- Uerscheln Johannes, Germany
- Ulmer Joachim, Germany
- Ulrich Thomas, Germany
- van Bodegom Peter, Germany
- Venske Wolf-Uwe, Germany
- Versen von Lutz-H, Germany
- Vismane Liana, Germany
- von Engelhardt Charlotte, Germany
- Voss-Dirks Hans-Peter, Germany
- Wagner Norbert, Germany
- Wallenfang Gero, Germany
- Waltert Mathias, Germany
- Wartner Bernhardt, Germany
- Wehgartner-Winkler Sabina, Germany
- Weimer Joachim, Germany
- Welss Christof, Germany
- Wende Wolfgang, Germany
- Wiedeking Burkhard, Germany
- Wiemer Silke, Germany
- Winkels Frank, Germany
- Winkler Joerg, Germany
- Wößner Rolf, Germany
- Wustmann Thomas, Germany
- Wuttke Wanda, Germany
- Zeisler Karl-Heinz, Germany
- Ziebuhr Susanne, Germany
- Zingler Wolfram, Germany
- Antoniadis Antonis, Greece
- Katsoulis Konstantinos, Greece
- Toumbis Michail, Greece
- Bálint Beatrix, Hungary
- Bánvölgyi Aranka, Hungary
- Böcskei Csaba, Hungary
- Csilla Szabó, Hungary
- Czompó Márta, Hungary
- Fabian Mariann, Hungary
- Kerenyi Ildiko, Hungary
- Márk Zsuzsanna, Hungary
- Papp Márta, Hungary
- Radeczky Éva, Hungary
- Rajkay Katalin, Hungary
- Sarosi Veronika, Hungary
- Schlezák Judit, Hungary
- Szolnoki Erzsébet, Hungary
- Casserly Brian, Ireland
- Fahy Ruairi, Ireland
- O'Callaghan Dermot, Ireland
- Adinolfi Luigi Elio, Italy
- Albergati Michele, Italy
- Ballarin Andrea, Italy
- Balzano Gianni, Italy
- Barbagallo Mario, Italy
- Bellofiore Salvatore, Italy
- Berra Daniele, Italy
- Bertocco Elisabetta, Italy
- Bianco Andrea, Italy
- Cardaci Vittorio, Italy
- Catapano Giosuè Angelo, Italy
- Cerini Giuseppe, Italy
- Ciccarelli Michele, Italy
- Corbi Graziamaria, Italy
- Corrao Salvatore, Italy
- Cosimo Franco, Italy
- Fedele Francesco, Italy
- Fiorentino Giuseppe, Italy
- Gibellino Maria Francesca, Italy
- Giorgio Vincenza, Italy
- Gulotta Carlo, Italy
- Idotta Giuseppe, Italy
- Launaro Nicola, Italy
- Lazzari Agli Luigi Arcangelo, Italy
- Mariotta Salvatore, Italy
- Martucci Michele, Italy
- Mastroberardino Michele, Italy
- Mazza Francesco, Italy
- Mirabella Saverio, Italy
- Monni Maria Cristina, Italy
- Morlino Paride, Italy
- Nardini Stefano, Italy
- Negrin Rolando, Italy
- Ortu Riccardo, Italy
- Papale Maria, Italy
- Papalia Antonella, Italy
- Pesci Alberto, Italy
- Picca Vito, Italy
- Pirina Pietro, Italy
- Pistelli Francesco, Italy
- Pistolesi Massimo, Italy
- Poletti Venerino, Italy
- Resta Onofrio, Italy
- Rottoli Paola, Italy
- Sabato Eugenio, Italy
- Sanduzzi Zampelli Alessandro, Italy
- Sarzani Riccardo, Italy
- Scarantino Pasquale, Italy
- Schillaci Giuseppe, Italy
- Sciarabba Catalano Giuseppe
- Filippo, Italy
- Simonassi Claudio, Italy
- Squillante Francesco, Italy
- Tantucci Claudio, Italy
- Tazza Roberto, Italy
- Torlasco Maurizio, Italy
- Torrini Alessandro, Italy
- Trevisan Fiorenza, Italy
- Tursi Francesco, Italy
- Vincenti Rigoletta, Italy
- Vincenzi Umberto, Italy
- Zambotto Franco Maria, Italy
- Zamprogna Claudio, Italy
- Aurika Babjoniseva, Latvia
- Kroica Iveta, Latvia
- Lilita Mitrofanova, Latvia
- Natalija Visocka, Latvia
- Puzule Sarmite, Latvia
- Reinholde Ilze, Latvia
- Vikmane Inga, Latvia
- Davoliene Inga, Lithuania
- Griskeviciene Violeta, Lithuania
- Kiziela Arturas, Lithuania
- Matukiene Violeta, Lithuania
- Sakalauskas Raimundas, Lithuania
- Vaicius Dalius, Lithuania
- Vaitkiene Nijole, Lithuania
- Vebriene Jolita, Lithuania
- Volosevic Teresa, Lithuania
- Båtevik Roy, Norway
- Holmberg Casper, Norway
- Høynes Kristian, Norway
- Karlsson Thomas, Norway
- Khorshidi Hossein, Norway
- Khusrawi Alamdar, Norway
- Norheim Paal, Norway
- Risberg Knut, Norway
- Solnør Leidulv, Norway
- Asankowicz-Bargiel Beata, Poland
- Chorostowska-Wynimko Joanna, Poland
- Dąbrowiecki Piotr, Poland
- Mróz Robert, Poland
- Napora Piotr, Poland
- Olechnowicz Dariusz, Poland
- Pulka Grażyna, Poland
- Waszkuć-Golonko Joanna, Poland
- Andre Sandra, Portugal
- Cardoso Joao, Portugal
- Catarina Guimarães, Portugal
- Drummond Marta, Portugal
- Ferreira Fernando, Portugal
- Gilberto Teixeira, Portugal
- Paula Rosa, Portugal
- Pires Nuno, Portugal
- Rodrigues Cidalia, Portugal
- Berlea Lavinia, Romania
- Macesanu Simona, Romania
- Mincu Viorica, Romania
- Montia Tatiana, Romania
- Savu Angelica, Romania
- Tofolean Doina, Romania
- Trailescu Ana Maria, Romania
- Ungureanu Dragos, Romania
- Abrosimov Vladimir, Russia
- Astafieva Natalia, Russia
- Barbarash Olga, Russia
- Gantseva Khalida, Russia
- Ignatova Galina, Russia
- Ilkovich Mikhail, Russia
- Irina Irkhina, Russia
- Nosov Vladimir, Russia
- Ovcharenko Svetlana, Russia
- Palyutin Shamil, Russia
- Punin Alexandr, Russia
- Shaporova Natalia, Russia
- Sinopalnikov Alexander, Russia
- Starovoitova Elena, Russia
- Trofimov Vasilii, Russia
- Vizel Alexander, Russia
- Zagidullin Shamil, Russia
- Babčáková Emília, Slovakia
- Frajtová Ľuboslava, Slovakia
- Gremeň Ľudovít, Slovakia
- Horváthová Helena, Slovakia
- Karakó Pavol, Slovakia
- Kavková Denisa, Slovakia
- Kováčiková Lucia, Slovakia
- Kubíková Yveta, Slovakia
- Leščišinová Helena, Slovakia
- Letonja Saša, Slovenia
- Lopert Anton, Slovenia
- Michaličková Miriam, Slovakia
- Plutinský Ján, Slovakia
- Šofranko Martin, Slovakia
- Szárazová Monika, Slovakia
- Žáčik Miroslav, Slovakia
- Klobučar Albert, Slovenia
- Košnik Mitja, Slovenia
- Alvarez Carlos Jose, Spain
- Álvarez Carmen, Spain
- Arzua Delfín, Spain
- Barrueco Miguel, Spain
- Cebrian Jose Joaquin, Spain
- Fernández Estrella, Spain
- Garcia Jose Ignacio, Spain
- García José Manuel, Spain
- Garcia-Salmones Mercedes, Spain
- Herrejón Alberto, Spain
- Jurado Bernabe, Spain
- Lores Luis, Spain
- Marin Alizia, Spain
- Mateos Caballero Luis, Spain
- Mayoralas Sagrario, Spain
- Melchor Rosario, Spain
- Mir Isabel, Spain
- Monsó Molas Eduard, Spain
- Montoliu Rosa, Spain
- Naval Sendra Elsa, Spain
- Ordoñez Xavier, Spain
- Otriz de Saracho Juan, Spain
- Palop Marta, Spain
- Riesco Miranda Juan Antonio, Spain
- Roca Josep, Spain
- Rodriguez Álvarez Mar, Spain
- Rodriguez Esther, Spain
- Roig Francisco José, Spain
- Romero Antonio, Spain
- Sala Ernest, Spain
- Sánchez Toril Fernando, Spain
- Serra Batles Joan, Spain
- Sevilla Ignacio, Spain
- Solanas Prats José Vicente, Spain
- Terns Manel, Spain
- Tolosana José María, Spain
- Toran Montserrat Pere, Spain
- Velasco Garrido José Luis, Spain
- Bleckert Jan, Sweden
- Curiac Dan, Sweden
- Gillblad Anders, Sweden
- Jaedig Steen, Sweden
- Leonardsson-Hellgren Margareta, Sweden
- Luts Anders, Sweden
- Nilsson Anna, Sweden
- Nilsson Folke, Sweden
- Romberg Kerstin, Sweden
- Sjöberg Folke, Sweden
- Sloma Andrzej, Sweden
- Adams Fran, UK
- Ainsworth Paul, UK
- Blagden Mark, UK
- Bodalia Bhavesh, UK
- Bundy Charlie, UK
- Cahill Tom, UK
- Clark Rebecca, UK
- Craze Andrew, UK
- Crouch Matthew, UK
- Davies Emyr, UK
- Dixon Peter, UK
- Durrant Gavin, UK
- Eavis Patrick, UK
- Feeroz Jaafar, UK
- Fuller Liz, UK
- Fullerton Duncan, UK
- Haddy Caroline, UK
- Hall Timothy, UK
- Harrison Richard, UK
- Heer Amardeep, UK
- Jackson Alan, UK
- Jackson-Voyzey Ewart, UK
- Jeffers Lesley, UK
- Kenyon Susan, UK
- Kerrane Jerome, UK
- Konig Dirk, UK
- Litchfield Jennifer, UK
- Logie Brian, UK
- Martin Ross, UK
- McCaldin Michael, UK
- McEleny Paul, UK
- Moorcroft Alexander, UK
- Naseem Gill, UK
- Pepperell Justin, UK
- Purohit Jaykumar, UK
- Reed Rory, UK
- Ryan John, UK
- Saralaya Dinesh, UK
- Sharma Rajiv, UK
- Simpson David, UK
- Smith Andrew, UK
- Symonds Rehan, UK
- Turner Wayne, UK
- Wakeling John, UK
- Waldron Michael, UK
- Wright Anthony, UK
